# HMGB1-Mediated Neuroinflammatory Responses in Brain Injuries: Potential Mechanisms and Therapeutic Opportunities

**DOI:** 10.3390/ijms21134609

**Published:** 2020-06-29

**Authors:** Yam Nath Paudel, Efthalia Angelopoulou, Christina Piperi, Iekhsan Othman, Mohd. Farooq Shaikh

**Affiliations:** 1 Neuropharmacology Research Strength, Jeffrey Cheah School of Medicine and Health Sciences, Monash University Malaysia, Bandar Sunway, Selangor 47500, Malaysia; Iekhsan.othman@monash.edu; 2 Department of Biological Chemistry, Medical School, National and Kapodistrian University of Athens, 11527 Athens, Greece; angelthal@med.uoa.gr

**Keywords:** high mobility group box 1 (HMGB1), traumatic brain injury (TBI), subarachnoid hemorrhage (SAH), neuroinflammation, biomarker

## Abstract

Brain injuries are devastating conditions, representing a global cause of mortality and morbidity, with no effective treatment to date. Increased evidence supports the role of neuroinflammation in driving several forms of brain injuries. High mobility group box 1 (HMGB1) protein is a pro-inflammatory-like cytokine with an initiator role in neuroinflammation that has been implicated in Traumatic brain injury (TBI) as well as in early brain injury (EBI) after subarachnoid hemorrhage (SAH). Herein, we discuss the implication of HMGB1-induced neuroinflammatory responses in these brain injuries, mediated through binding to the receptor for advanced glycation end products (RAGE), toll-like receptor4 (TLR4) and other inflammatory mediators. Moreover, we provide evidence on the biomarker potential of HMGB1 and the significance of its nucleocytoplasmic translocation during brain injuries along with the promising neuroprotective effects observed upon HMGB1 inhibition/neutralization in TBI and EBI induced by SAH. Overall, this review addresses the current advances on neuroinflammation driven by HMGB1 in brain injuries indicating a future treatment opportunity that may overcome current therapeutic gaps.

## 1. Introduction

Traumatic brain injury (TBI) is a devastating disorder associated with a major cause of morbidity and mortality leading to significant direct and indirect costs to society. TBI refers to a complex disorder associated with several degrees of contusion, diffuse axonal injury (DAI), hemorrhage, and hypoxia [1]. These effects cumulatively initiate biochemical and metabolic changes causing gradual tissue damage and cell death [2]. Although several agents have shown promising neuroprotective effects against TBI in pre-clinical settings, they failed to improve outcome in clinical trials [3,4]. These poor effects may be attributed to the disparate nature of TBI, with differences in clinical variables such as tissue biomechanics site and severity of injury [5,6].

Despite the extensive advances in clinical and pre-clinical research, the pathomechanism underlying TBI are still elusive. However, there is evidence on the contribution of neuroinflammation and blood–brain barrier (BBB) disruption in driving the secondary damage after brain injury [7]. Improved neurological effects have been reported in experimental TBI upon inhibition of post-traumatic neuroinflammatory response [8,9].

Targeting neuroinflammation to inhibit the secondary damage post-TBI represents an effective strategy in recent days. One of the inflammatory mediators that seem to be implicated in brain injury is the high mobility group box 1 (HMGB1) protein, a pro-inflammatory-like cytokine released actively after activation of other cytokines and passively during cell death [10]. HMGB1 released from stressed and dying brain cells acts as an effective neuroinflammatory mediator [11]. HMGB1 is mainly expressed in all cell types, including neurons and glial cells. Overexpression of extracellular HMGB1 was observed in several neuroinflammatory conditions, including TBI [12], Subarachnoid hemorrhage (SAH) [13], Epilepsy [14], Alzheimer’s diseases (AD) [15], Amyotrophic lateral sclerosis (ALS) [16,17] Parkinson’s diseases (PD) [18], etc.

Furthermore, HMGB1-mediated neuroinflammatory response has been implicated in several types of brain injury including TBI [19] and early brain injury (EBI) after SAH [13] as well as in cerebral ischemia-induced and hypoxic ischemic (HI)-induced brain injuries [20].

**Figure 1:**
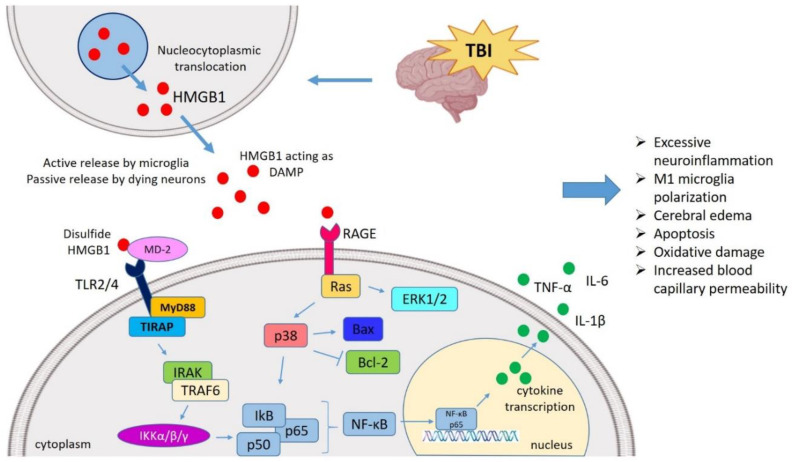
HMGB1 mediated neuroinflammatory response in TBI. TBI induces nucleocytoplasmic translocation of HMGB1 resulting into the release of HMGB1 in extracellular milieu. The extracellular HMGB1 may be partially oxidized at the two cysteine residues generating the disulfide form of HMGB1. The disulfide HMGB1 further binds to its prominent receptor system such as TLR4 and RAGE which in turn interacts with MD-2 and initiates the MyD88 dependent pathway. It also binds to Ras to initiate the ERK pathway, respectively. HMGB1-TLR4 axis can activate NF-κB signaling both directly and through TRAF6. These pathways ultimately interact with the NF-κB lead to the generation of neuroinflammatory response by producing several pro-inflammatory cytokines (TNF-α, IL-1β, and IL-6). In this way, HMGB1 might mediate the TBI-induced secondary injury where HMGB1 is understood to amplify vicious neuroinflammation, M1 polarization, apoptosis, oxidative damage, cerebral edema, increased BBB permeability. TBI, Traumatic brain injury; HMGB1, High mobility group box 1; TLR4, Toll-like receptor 4; MD-2, Myeloid differentiation factor-2; MyD88, Myeloid differentiation response protein 88; ERK, Extracellular signal-related kinase; NF-κB, Nuclear factor κ light chain enhancer of activated B cells; TNF-α, Tumor necrosis factor-α; IL, Interleukin; BBB, Blood–brain barrier; TRAF6, TNF receptor-associated factor 6.

HMGB1 was demonstrated to drive the neuroinflammatory response after TBI leading to secondary damage as evident by its up-regulated expression and release after experimental TBI induction (Figure 1) [21,22]. Similarly, HMGB1-mediated neuroinflammatory response contributes to early brain injury (EBI) after SAH [13]. Active HMGB1 translocation from nuclei to the extracellular space after TBI and SAH was shown to activate neuroinflammatory cascades and rupture the BBB. Of interest, HMGB1-targeted therapies such as an anti-HMGB1 monoclonal antibody (mAb) and the pharmacological inhibitor Glycyrrhizin were effective experimentally in inhibiting the neuroinflammatory response post-TBI and in minimizing the EBI after SAH. This reflects that HMGB1 might be a promising extracellular target against these conditions leading to new treatment opportunities.

Herein, we address the role of HMGB1-mediated neuroinflammatory response as a mechanism of secondary injury after TBI and HMGB1-mediated neuroinflammation in EBI after SAH. Moreover, we discuss the emerging preclinical findings of HMGB1-targeting studies demonstrating inhibition of both TBI-induced secondary damage and EBI after SAH.

## 2. Insights into the HMGB1 Biology

HMGB1 is a 25-kDa DNA-binding protein composed of 215 amino acids, consisting of two DNA bindings sites (Box A and Box B) and a negatively charged *C*-terminal [23,24]. HMGB1 can be secreted extracellularly through active and passive release that occurs in both somatic and immune cells [25]. After reaching to the extracellular milieu, HMGB1 serves as a damage-associated molecular pattern (DAMP) protein and exerts a compelling inflammatory response, engaging several inflammatory mediators. Upon release from neurons and astrocytes, HMGB1 begins the production of several inflammatory markers, including TNF-α, IL-6, and IL-1β [26]. Extracellular HMGB1 interacts with several pathogen recognition receptors (PRRs), namely the toll-like receptor 4 (TLR4) and the receptor for advanced glycation end products (RAGE), to initiate cell migration and production of cytokines [27]. The ability of HMGB1 to interact with several receptors depends on the post-translational redox modifications (PTMs) of the three cysteine residues (at positions 23, 45, and 106 in the box A and B domains of HMGB1) [28].

Moreover, three isoforms of HMGB1 have been identified: the fully reduced HMGB1, the disulfide HMGB1, and the sulphonyl HMGB1. The fully reduced isoform of HMGB1 binds to CXCL12, which further interacts with a great affinity to CXCR4. The extracellular TLR4 adaptor myeloid differentiation factor-2 (MD-2) forms a complex only with the disulfide isoform of HMGB1 (not to any other redox forms) and aggravates the expression of chemokines and cytokines [29] at a comparable level to lipopolysaccharide (LPS)-induced production of pro-inflammatory cytokines [30]. Disulfide HMGB1 signaling through TLR4 enhances the activation of nuclear factor-κ light chain enhancer of activated B cells (NF-κB) and consequently the transcription of pro-inflammatory cytokines [31]. Due to its cytokine-inducing potential, HMGB1 has gained increased attention in recent days and was extensively studied in several inflammatory diseases [23].

## 3. HMGB1-Mediated Neuroinflammatory Response in TBI

TBI represents a complicated and heterogeneous condition caused by an external direct or indirect mechanical effect on the brain, resulting in the disruption of the physiological brain structure and function [32]. The severity of TBI depends mainly on the level of consciousness and is clinically evaluated by the Glasgow Coma Scale (GCS), which categorizes TBI severity into mild, moderate, or severe [33]. TBI is a leading cause of mortality and morbidity, accounting for 2,500,000 cases in the EU and 3,500,000 in the US, per annum [34]. Despite extensive research efforts in the field of brain trauma as well as the praiseworthy progress in clinical diagnostics and management, there are still no effective neuroprotective strategies to alleviate the neurotoxic pathways exacerbating the initial injury [35].

The primary insult in TBI leads to a secondary injury that is mediated by the positive feedback cascades of neuroinflammation, cell death, disruption of BBB, and glutamatergic excitotoxicity. All the components of this feedback loop lead to aggravation of neuronal dysfunction and tissue loss [36]. More precisely, TBI-mediated phenomena of secondary injury include enduring neuroinflammation over time that may contribute to chronic neurological impairment [37]. The delayed nature of neuroinflammation reflects the possibility of a therapeutic window for intervention against the progressive brain tissue damage and the subsequent improvement of functional recovery after injury [38].

HMGB1 has been significantly associated with the detrimental effects of brain injury in several traumatic and non-traumatic brain injury models. It has been shown to increase the chemotaxis and activation of leukocytes ex vivo [31,39,40]. It further triggers microglial activation, enhances neuroinflammation, and subsequently exacerbates neurocognitive impairment, at least partially in a TLR-dependent manner in several models of non-traumatic injury [41,42,43]. TBI-induced microglial activation and increased expression of inflammatory mediators (HMGB1, TNF-α, IL-1β, IL-6) in the brain have been associated with cerebral edema and neurological deficits [44]. NR2B-mediated release of HMGB1 from neurons undergoing necrosis promotes post-TBI brain edema and correlates with the increased intracranial pressure (ICP) observed in human patients. The damaging effects of HMGB1 were mediated, at least partially by TLR4 stimulation in microglia and a gradually increased expression of aquaporin-4 (AQP4) [45].

The HMGB1 protein has been studied extensively in pre-clinical TBI models where it can interact with TLRs and activate mitogen-activated protein kinase (MAPK) and NF-κB pathway, resulting in the excessive release of multiple pro-inflammatory cytokines, such as TNF-α, IL-1β, and IL-6 (Figure 1) [46]. Notably, these factors might affect the local environment of the brain after CNS injury and exacerbate brain damage after TBI [47], therefore suggesting that the modulation and targeting of inflammatory responses after TBI may represent a promising therapeutic strategy in minimizing the secondary damage [48].

In a rat model of TBI, HMGB1 expression was significantly reduced below its basal levels at 6 h after TBI, whereas it gradually reached basal levels at 2 days after the insult. Similarly, RAGE expression increased at 6 h and reached its peak levels at 24 h after the insult, whereas they were gradually decreased and maintained a higher expression than those of the sham group at day 6 [19]. The observed expression of HMGB1 and RAGE after TBI indicate the contribution of HMGB1 in the post-TBI secondary inflammatory process. Although HMGB1 is known to interact with the two principal receptors RAGE and TLR4 [49] RAGE seems to be the main mediator of the effects of HMGB1 in the case of TBI [12].

Interestingly, an age-dependent release of HMGB1 was demonstrated in controlled cortical impact (CCI)-induced experimental models of pediatric (3 weeks) and adult (8–10 weeks) TBI. More specifically, after TBI, extracellular HMGB1 has been increased in both lesional and perilesional neocortex of the pediatric and adult mice, compared to sham controls. However, the elevation of HMGB1 was statistically significant only in the perilesional neocortex of adult mice, suggesting that TBI-induced neuronal damage may be accompanied by release of HMGB1 into the extracellular milieu. Regarding circulating HMGB1 levels, a fluctuation in the concentration of serum HMGB1 was observed in pediatric TBI, whereas it remained relatively steady in adult TBI [22]. These findings suggest that the growing pediatric brain may represent a better candidate for therapeutic targeting/inhibition of HMGB1 to prevent the harmful effects of TBI-induced neuroinflammation, in comparison to the adult brain [22].

Moreover, microglia are well-established as the primary mediators of the innate inflammatory responses in the brain [50]. They have been shown to be rapidly activated at the site of the insult and differentiate either into a detrimental/pro-inflammatory (M1) or a beneficial/anti-inflammatory (M2) phenotype, based on the specific conditions of their host tissue microenvironment [51,52]. In the case of TBI, although a combination of both M1 and M2 phenotypes has been reported, M1 predominates over the M2 microglial phenotype [51,53,54]. Previous pre-clinical studies have demonstrated that by inhibiting the activation of the M1 phenotype and increasing the activation of the M2 phenotype, the neuronal damage was ameliorated and the functional recovery after TBI was enhanced [55,56], suggesting the modulation of microglia/macrophage polarization as a novel therapeutic strategy. HMGB1 has been shown to activate macrophages via TLR4, TLR2, and RAGE/NF-κB signaling cascades, and induce the polarization of macrophages into M1 phenotype [57]. In an experimental TBI study, treatment with the natural HMGB1 inhibitor, glycyrrhizin could improve the functional recovery, decrease the lesion volume, suppress the HMGB1 expression and release, as well as reduce the activation of M1 phenotype while promoting the activation of M2 phenotype of microglia/macrophages after TBI [58]. These findings suggest that glycyrrhizin may be able to ameliorate TBI-induced neuronal damage by promoting the polarization of microglia/macrophages into the anti-inflammatory M2 phenotype, at least partially via HMGB1 inhibition.

The contribution of HMGB1 in the secondary post-TBI damage is based on the studies that report an up-regulated expression of HMGB1 in experimental TBI. Moreover, HMGB1 has emerged as a potential therapeutic target against TBI with promising outcomes [12,21,59].

Increasing evidence indicates that extracellular release of protein by post-traumatic necrotic cells can induce inflammatory responses and subsequent cerebral edema by up-regulating the expression of RAGE and TLR4, as well as by reducing the levels of BBB-associated proteins ZO-1, Occludin, and Claudin-5. Moreover, the post-traumatic increase of HMGB1 has been shown to negatively affect other secondary post-traumatic damage processes by promoting apoptosis, as shown by increased Bax protein and caspase-3, and decreased Bcl-2. In respect to the relationship between post-traumatic HMGB1 up-regulation and oxidative stress, a reduced total antioxidant status (TAS) and increased total oxidant status (TOS) along with elevated oxidative stress index (OSI) have been observed [60].

In a fluid percussion injury (FPI)-induced pre-clinical model of TBI, a significant increase in HMGB1 expression has been reported, which was, in turn, down-regulated after treatment with anti-HMGB1 monoclonal antibodies (mAbs). Moreover, anti-HMGB1 mAb treatment has been shown to suppress acute brain edema, as assessed by magnetic resonance imaging (MRI), protect the permeability of BBB, inhibit the expression of pro-inflammatory molecules and improve motor function when evaluated by rotarod test [12].

However, the findings of a recent study investigating the effects of HMGB1 deletion in a knockdown (KO) CCI-induced TBI model support the hypothesis that HMGB1 contribution in TBI is rather a “double-edged sword”. In particular, HMGB1 KO does not prevent brain edema, as well as does not protect BBB permeability, hippocampal neuronal survival (CA1 and CA3 regions of the contralateral, and ipsilateral hemispheres) and hemispheric tissue loss, in comparison to the wild type (WT) mice [61]. These findings highlight the need for further and deeper exploration of the role of HMGB1 in TBI, as well as for careful selection of HMGB1 blocking agents/strategies (degree, location, and duration) to develop novel effective treatment schemes.

## 4. HMGB1 Translocation Post-TBI: Insights from Experimental Findings

Under pathological conditions, HMGB1 can be up-regulated, translocated from the nucleus into the cytoplasm, and secreted by activated microglial cells, acting as a pro-inflammatory factor [62]. It is well-established that HMGB1 can be transferred from the nucleus to cytoplasm in the injured site of various HMGB1-mediated pathologies, such as TBI [59], epilepsy [14], AD [63] and PD [18].

After TBI, time-dependent localization of HMGB1 is detected by immunohistochemical analysis. HMGB1 staining was absent in the center of the contused area at 30 min post-TBI, whereas it was widely expressed in the nuclei of the sham-injury group. Moreover, HMGB1 expression has faded at 1-h post-TBI and it was found sub-localized inside the cytosol of neuronal cells (contused area) at 2 h post-TBI. Furthermore, HMGB1 staining was detected in the cytosol of glial cells at 4 h after TBI [19].

Nucleocytoplasmic translocation and extracellular release of HMGB1 are crucial events of TBI-induced activation of microglia and the following inflammatory responses [45]. In the fluid percussion-induced (FP) TBI model, a nuclear-cytoplasmic translocation of HMGB1 has been demonstrated in the microtubule-associated protein 2 (MAP2)-positive neurons, mainly in the hippocampus and cerebral cortex. However, no translocation was observed in glial fibrillary acidic protein (GFAP)-positive astrocytes or ionized calcium-binding adapter molecule 1 (Iba1)-positive microglia at the site below the percussion at 6 h after the insult in control rats. Moreover, some neuronal cells in the hippocampus and cerebral cortex displayed HMGB1-immunoreactive granule-like structures in the cytoplasm at 6 h after injury, whereas HMGB1 immunoreactivity (IR) decreased in other neuronal cells at the primary lesion site [59].

In a Feeney DM TBI model, there was a significant expression of HMGB1 at 3 days post-injury, in neurons, Iba-1-positive microglia, and GFAP-positive astrocytes, as evaluated by double immunofluorescent staining. Moreover, elevated expression of HMGB1 was observed in the cytosol and nucleus of both neuronal cells and microglia from the injured cortices, as assessed by western blot analysis. The reduction of HMGB1 translocation and release further resulted in down-regulation of the NF-κB pathway and attenuation of the post-TBI inflammatory responses [44]. HMGB1 was mainly distributed in the nucleus, whereas fewer TLR4- and RAGE-positive cells were observed in the sham group. However, following its nucleocytoplasmic translocation, HMGB1 was mainly redistributed in the cytoplasm of the experimental TBI model induced by the modified Feeney method [64]. HMGB1 was widely localized in the neocortex (cortical pyramidal neurons) and hippocampus (DG and CA1) of the uninjured rats. On the contrary, 3 days post-TBI, HMGB1 cell staining was accompanied by an increased extracellular HGMB1 staining, and fewer NeuN^+^ neurons were observed in the lesion core, which lacked HMGB1 labeling.

Collectively, the abovementioned findings suggest that neuronal damage may be associated with HMGB1 translocation into the extracellular space after TBI (pediatric and adult). Moreover, the investigation of HMGB1 protein levels as well as nuclear and cytosolic fractions, revealed increased total protein levels of HMGB1 at 3 days after TBI, compared to sham groups. Increased levels of cytosolic and nuclear HMGB1 were detected at 2, 6 and 24 h, as well as at 3 and 7 days after the insult in adult rats (except for cytosolic fractions 7 days after the injury). On the contrary, no alterations in HMGB1 protein levels (total, cytosolic or nuclear) were identified after injury in pediatric cases [22].

Although it is evident that HMGB1 can translocate after TBI, the expression of HMGB1 after TBI was different among the reported studies. More specifically, some studies have demonstrated a reduction in HMGB1-expressing cells in the first few days after the injury [19], whereas others showed an up-regulation of HMGB1 levels at 24 h after the insult, restricted to the HMGB1 cytosolic fraction and reflecting the nucleocytoplasmic translocation of HMGB1 [65].

Discrepancies in HMGB1 release patterns among different studies could be attributed to the nature of the injury since more severe or diffuse brain insults could result in increased HMGB1 release from damaging neurons and/or nuclear to cytosol translocation [22].

## 5. HMGB1 as a Potential Biomarker to Predict Outcome after TBI

Since therapeutic strategies against TBI aim mainly to minimize the secondary damage after the injury, a novel biomarker able to reflect this secondary damage is undoubtedly needed. In this regard, HMGB1 protein may represent a promising biomarker, which could not only predict disease progression but also provide an insight into the therapeutic outcome. Of note, the prognostic value of HMGB1 has been similar to that of GCS scores, highlighting its emerging biomarker potential in TBI [66].

A clinical study indicated that increased levels of HMGB1 in ventricular CSF were correlated with poorer outcome after TBI in children. Moreover, peak HMGB1 levels were demonstrated to be inversely and independently correlated with the Glasgow Outcome Scale (GOS) scores at 6 months after TBI [67].

HMGB1 was undetected in the CSF of normal pressure hydrocephalus (NPH) patients, whereas high expression of HMGB1 was shown in the CSF of TBI patients, suggesting that HMGB1 release may be driven by trauma, and not by increased hydrocephalus [45], supporting the biomarker potential of HMGB1 in the CSF of TBI cases and highlighting its specificity compared to other neurological disorders.

Furthermore, HMGB1 levels in the peripheral blood may be well correlated with disease outcome in TBI patients. Higher plasma concentrations of HMGB1 in TBI patients were detected in comparison to healthy controls on admission. Increased HMGB1 levels in the periphery were also detected in patients who died or suffered from the unfavorable outcome, a year after the brain injury. Moreover, HMGB1 levels on admission were greater compared to those of survivors or those with favorable outcomes [66].

Furthermore, in post-mortem brain tissues of TBI, patients’ translocation of HMGB1 was observed while it was localized in the nucleus of most cells in tissue sections of the controls. On the contrary, HMGB1 staining was either lost from the nucleus or sub-localized in the cytoplasm of cells in the contused area between 30 min and 1 day after the insult. It was localized and expressed in the cytoplasm of phagocytic microglia in the contused area between 2–20 days post-TBI [19].

Therefore, the strong implication of HMGB1 in the process of BBB disruption, TBI-induced neuronal injury, and activation of inflammatory response accounts for its great translational potential as a diagnostic and/or prognostic biomarker. The growing number of findings in recent years reporting an up-regulated expression of HMGB1 (serum and CSF) after TBI suggest that HMGB1 may represent a plausible biomarker (prognostic/diagnostic) to predict secondary damage after TBI, or to monitor neuroprotective drug efficacy against TBI in the future (Table 1).

On a limiting part, there is an increased understanding that average time for the laboratory estimation of circulating HMGB1 levels is comparatively higher whereas head tomographic images can be viewed within 10 min and the GCS scores can be made immediately available upon physical examination [66]. Similarly, an earlier study has raised a concern regarding the sensitivity and specificity in the use of CSF levels of HMGB1 as a prognostic biomarker [67]. Of note, the levels of HMGB1 detected by enzyme-linked immunosorbent assay (ELISA) technique do not precisely differentiate between the HMGB1 that is actively and passively released into the extracellular settings. Hence, the levels of HMGB1 detected by ELISA might depict necrotic cell death, or immunomodulatory release of HMGB1 from the macrophages and monocytes, or a combination of both [67]. This limitation can be overcome to some extent via the use of two-dimensional gel electrophoresis followed by immunoblotting where the HMGB1 that is actively released is hyper-acetylated [62]. These data reflect the pressing need for further investigation assessing the benefits of using HMGB1 as a plausible biomarker for TBI.

## 6. HMGB1 Mediated Neuroinflammation in Early Brain Injury (EBI) after SAH: Insights from Preclinical Findings

SAH is a devastating disease of the CNS affecting around 22.5 per 100,000 population [68], been associated with high mortality [69]. EBI and cerebral vasospasm are acknowledged as the two major complications after SAH, commonly occurring within 72 h and presenting the main reason behind the poor outcome [70]. Neuroinflammation is considered to be a crucial pathological phenomenon in EBI after SAH [71,72] along with other pathophysiological mechanisms such as elevated ICP, reduced perfusion pressure, disrupted BBB, brain ischemia and edema which may ultimately lead to neuronal injury and death [73].

**Figure 2:**
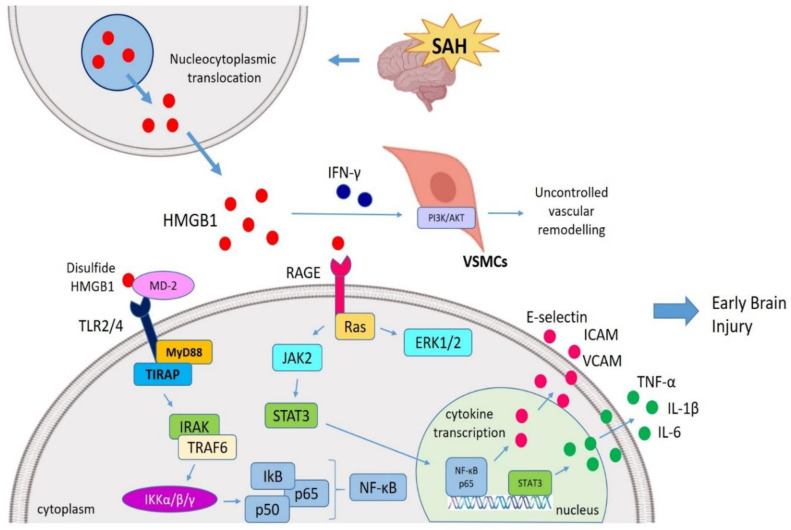
HMGB1-mediated EBI post-SAH. During SAH, after a HMGB1 translocation from nucleus to cytosol, extracellular HMGB1 interacts with TLR4 (via MD-2) and RAGE (via Ras) and initiates the MyD88 and JAK pathways. HMGB1 is also able to stimulate ERK1/ERK2 pathways. HMGB1 drives an inflammatory cascade via the JAK-STAT signaling pathway following SAH which ultimately resulted in the translocation of NF-κB p-65 to the nucleus to activate transcription of pro-inflammatory genes (IL-1β and TNF-α). In addition, HMGB1 also amplifies the secretion of VCAM, ICAM and E-selectin. In this regard, HMGB1 mediate the early brain injury (EBI) followed SAH as well as vascular remodeling post-SAH (mainly by triggering VSMCs via IFN-γ interaction). SAH, Subarachnoid hemorrhage; HMGB1, High mobility group box 1; TLR4, Toll-like receptor 4; RAGE, Receptor for advanced glycation end products; MD-2, Myeloid differentiation factor-2; MyD88, Myeloid differentiation response protein 88; ERK, Extracellular signal-related kinase; JAK, Janus kinase; NF-κB, Nuclear factor κ light chain enhancer of activated B cells; EBI, Early brain injury; VSMCs, Vascular smooth muscle cells; VCAM, Vascular cell-adhesion molecule; ICAM, Intercellular adhesion molecule.

Increased expression of extracellular HMGB1 after SAH has been shown to aggravate inflammation and trigger the up-regulation of downstream inflammatory factors via TLRs/NF-κB and RAGE/NF-κB signaling cascades. In turn, up-regulated inflammatory mediators further increase HMGB1 expression, leading to HMGB1 translocation from the nucleus to the extracellular milieu. HMGB1 has been proposed to regulate the damaging inflammatory response and may serve as a key contributor to the inflammatory process underlying SAH (Figure 2) [74].

HMGB1 expression is increased post-SAH, and is implicated in its pathogenesis [75]. HMGB1 contributes to the SAH-induced EBI mainly by activating Janus kinases 2 (JAK2)/Signal transducer and activator of transcription (STAT3). In an in vivo SAH model induced by endovascular perforation, total HMGB1 protein was shown up-regulated along with phosphorylation of JAK2/STAT3. In more detail, cytosolic HMGB1 post-SAH was initially increased and then gradually decreased, whereas nuclear HMGB1 was initially reduced and then elevated after SAH [76]. Upon inhibition of the phosphorylation of JAK2/STAT3 by the JAK2/STAT3 inhibitor AG490, the up-regulated expression and translocation of HMGB1 was reduced and EBI was ameliorated, further highlighting the contribution of JAK2/STAT3 pathway in HMGB1-mediated neuroinflammation in SAH [76].

Additional evidence indicates that the expression of HMGB1 is increased immediately after SAH. In the experimental SAH model induced by prechiasmatic injection, HMGB1 protein increased at 2 h post-SAH and reached its maximum peak concentration after 1 day, compared to sham controls. These findings imply that SAH may lead to an early up-regulated production and translocation of HMGB1 protein in the brain [77]. Furthermore, extracellular HMGB1 has been shown to exert a pro-inflammatory role and contribute to brain injury after SAH, since TLR4, IL-1β, and NF-κB (pro-inflammatory subunit P65) expression was up-regulated after the administration of recombinant-HMGB1 [77].

HMGB1 has been also reported to trigger inflammation and contribute to vascular remodeling or repair after SAH. It has been shown to increase the secretion of vascular cell-adhesion molecule (VCAM), intercellular cell-adhesion molecule (ICAM), as well as E-selectin during SAH [78]. Upon binding to TLR4/TLR2 and RAGE, HMGB1 has been demonstrated to amplify the inflammatory cascades leading to activation of NF-κB, IL-6, IL-8, TNF-α, MyD88, iNOS, extracellular-regulated kinase 1 (ERK1) and ERK2 [79]. HMGB1/RAGE interaction is crucial for neural function recovery during the post-SAH recovery period. Along with HMGB1, there is also an up-regulation of RAGE expression in the injured brain cortex, 14 days after SAH, in comparison to sham controls, pointing to a potential effect of HMGB1 in the recovery of brain damage after SAH [80].

Furthermore, HMGB1 was shown to interact with IFN-γ to trigger the vascular smooth muscle cells (VSMCs) and regulate VSMC phenotype switching during SAH [81]. Modulation of Phosphoinositide 3-kinases/protein kinase B (PI3K/AKT) pathway has been shown to lead to VSMC proliferation, resulting in uncontrolled vascular remodeling [82]. Upon treatment with anti-HMGB1 mAb, this vascular remodeling and VSMC phenotypic switching observed in SAH was found to be attenuated, mainly by reversing SAH-induced up-regulation of HMGB1, microglial activation and brain edema [81].

A better understanding of the mechanisms underlying EBI may help toward the development of novel treatment strategies to improve the clinical outcome after SAH. Targeting HMGB1-mediated signaling pathways may represent a promising future therapeutic strategy. Nevertheless, a more comprehensive approach is rather crucial to partially inhibit HMGB1 pro-inflammatory effects, without altering its beneficial mechanisms that underlie neurovascular remodeling [80].

## 7. Evidence of HMGB1 Translocation Post-SAH: Evidence from Experimental Studies

Translocation of HMGB1 from the nucleus might aggravate the neuroinflammatory response after SAH. Moreover, after its release to the extracellular milieu, HMGB1 can interact with TLR4 and lead to the up-regulation of the NF-кB pathway with the generation of pro-inflammatory mediators that aggravate SAH-induced brain injury [77,83]. Neuronal release of HMGB1 might prompt surrounding glial cells to generate inflammatory mediators that could, in turn, accelerate the neural cells to release more HMGB1 by elevating the HMGB1 mRNA levels [77]. Apart from neurons, microglial cells may present an additional source of HMGB1, thus contributing to increased HMGB1 expression and release after SAH [75,84].

In a prechiasmatic injection-induced SAH rat model, increased expression and cytoplasmic translocation of HMGB1 was observed in the cortex, 2 h after SAH, as evaluated by western blot analysis. Moreover, an increased amount of cytosolic HMGB1-positive cells in NeuN, Iba1 (resident microglia marker) and GFAP (astrocytic marker) positive cells has been detected by immunofluorescent staining, thus suggesting that HMGB1 translocation may occur mainly in neuronal cells of the injured cortex after SAH. During HMGB1 translocation, both passive release and active secretion of HMGB1 were observed. The translocated HMGB1 into the extracellular space induces further the expression of pro-inflammatory cytokines [77]. Immunohistochemical analysis has shown that a high percentage of cells (90%) which were positive for Iba1, were also positive for cytoplasmic HMGB1, indicating an enhanced cytosolic expression of HMGB1 in the brain parenchyma of the animals 5 days after SAH induction [75].

Earlier, HMGB1 was mainly expressed in the nucleus whereas no HMGB1 expression was in the cytosol of the normal (sham group) brain. The HMGB1 transcriptional expression was initiated at 6 h after SAH and at day 1 and 3 post-SAH there was a significant increase in the number of HMGB1 positive immuno-stained cells compared to the sham group [76]. In a modified prechiasmatic SAH model, immunofluorescence analysis showed localization of HMGB1 in neuronal nuclei at the frontal brain tissue of the control group, whereas HMGB1 was translocated into the cytoplasm after SAH induction [85].

Not only HMGB1 but also a total and cytosolic expression of TLR4 increased after SAH, as assessed by western blot analysis [86], suggesting that reducing cytoplasmic HMGB1 translocation in neurons might be a promising strategy. HMGB1 was further shown to be translocated from the smooth muscle cells into the basilar artery (BA) of SAH model rats. The dense HMGB1 immunoreactivity in the nuclei of VSMC of the BA in sham controls was depleted at 48 h after SAH, providing further evidence of the extracellular HMGB1 translocation in VSMCs [13].

Therefore, it is evident that the release of HMGB1 after SAH may differ, providing a time window for therapeutic intervention to prevent brain injury after SAH.

## 8. Biomarker Potential of HMGB1 to Predict EBI after SAH

HMGB1 has not only emerged as a crucial contributor to brain injury after SAH, but it also exhibits a biomarker potential for assessing the functional outcome. Elevated HMGB1 levels in experimental SAH models [87] suggest that increased HMGB1 expression may further aggravate the inflammatory responses after SAH.

The biomarker potential of HMGB1 has been mainly supported by the clinical findings where the up-regulated peripheral blood concentration was correlated with a poor outcome in aneurysmal SAH [88]. Reduction in mRNA and protein expression of HMGB1 upon treatment with the natural HMGB1 inhibitor Glycyrrhizin was shown to improve neurological scores in an experimental model of SAH [85].

Accumulated findings support the biomarker potential of HMGB1 in SAH since up-regulation in the expression levels of HMGB1 (plasma, CSF) was correlated with poor functional outcome (Table 2), suggesting HMGB1 as a possible marker of neuronal injury. Although these clinical findings reflect the contribution of HMGB1 in the pathogenesis of SAH-induced brain injury, contradictory data show no correlation between plasma HMGB1 levels and neurological effects in patients with aneurysmal SAH [89] and need further investigation.

## 9. HMGB1 Targeted Therapies against Brain Injuries

In recent days, HMGB1 has emerged as an extracellular target against a diverse range of CNS disorders with HMGB1 involvement, including AD [15], PD [18], MS [96], Epilepsy [97], TBI [12], SAH [13], etc. Moreover, HMGB1 targeted therapy conferred neuroprotective effects against these diseases mainly by inhibiting its expression and release, blocking its translocation, and down-regulating the expression of inflammatory molecules. Common HMGB1 targeting strategies in experimental studies include the use of anti-HMGB1 mAb and the natural HMGB1 inhibitors Glycyrrhizin with its derivatives, and Ethyl pyruvate [98,99] which have recently gained increasing attention as potential therapeutic strategies against several diseases of CNS and of the peripheral nervous system (PNS) [100,101]. In the following sections, the promising outcomes of HMGB1 inhibition by the anti-HMGB1 mAb and Glycyrrhizin in TBI and SAH are discussed.

### 9.1. HMGB1 Neutralization against TBI

In FPI-induced TBI, treatment with anti-HMGB1 mAb was shown to significantly suppress the HMGB1 translocation in neurons and retain its immunoreactivity in the nuclei. The anti-HMGB1 mAb attenuated neuronal cell death as evidenced by the intact nissl-positive pyramidal neurons and protected BBB integrity based on the significant inhibition of Evans blue leakage (by 88%), impeding the leakage area to the primary lesion area. Moreover, treatment with anti-HMGB1 mAb improved motor functions as evident from the rotarod and cylinder test and suppressed the activation of inflammatory molecules (TNF-α, iNOS, HIF-1α, COX-2, VEGF-A_189_, and VEGF-A_165_) [12].

The anti-HMGB1 mAb treatment in the FPI-induced TBI model also suppressed microglia as evident by reduced CD68-positive cells, ameliorated neuronal cell death in the hippocampus based on low TUNEL positivity, inhibited HMGB1 translocation and reduced plasma HMGB1 levels. Moreover, anti-HMGB1 mAb exerted beneficial effects on the impairment of motor and cognitive functions that were present for 14 days post-TBI [102].

Similarly, in a model of intracerebral hemorrhage (ICH)-induced brain injury, the anti-HMGB1 mAb inhibited its translocation into the extracellular space, decreased its serum levels and reduced brain edema while it maintained the permeability of BBB. Moreover, the anti-HMGB1 mAb reduced microglial activation, reduced oxidative stress, ameliorated behavioral performance and down-regulated the expression of inflammation-related mediators (TNF-α, iNOS, IL-1β, IL-6, IL-8R, COX-2, MMP2, MMP9 and VEGF 121) [103]. All these promising findings indicate that anti-HMGB1 mAb therapy might be beneficial against TBI.

The natural small molecule Glycyrrhizin binds directly to both HMG boxes, inhibiting its chemoattractant and mitogenic properties [98] and exerting protective effects against experimental TBI. Glycyrrhizin treatment ameliorated TBI-induced brain edema and beam walking distance, inhibited the translocation of HMGB1 and down-regulated TBI-induced up-regulation of HMGB1, RAGE, TLR4, and NF-κB. It further inhibited cell apoptosis and reduced TBI-induced up-regulation of inflammatory cytokines (TNF-α, IL-1β, IL-6) [64]. Glycyrrhizin conferred neuroprotection in a preclinical model of pediatric TBI by decreasing brain HMGB1 levels, edema, and preventing impairment of spatial and motor learning [21]. Thus, targeting HMGB1 by Glycyrrhizin might reduce the inflammatory cascades and ameliorate the motor function after TBI.

Please note that binding of Glycyrrhizin to HMGB1 is concentration-dependent with an equilibrium dissociation constant (Kd) value of 4.03 µM. Glycyrrhizin blocks HMGB1 binding to its receptor RAGE and inhibits TBI. In turn it inhibits the TBI-induced HMGB1 translocation and suppresses the reduction of HMGB1 from the site of injury. It further inhibits the BBB permeability and down-regulates the expression of inflammatory molecules (TNF-α, IL-1β, IL-6) [59]. It is worth noting that Glycyrrhizin has a wide therapeutic window, as shown by the fact that it is effective even at 6 h post-TBI [59].

It further affects microglial polarization, exerting a neuroprotective effect. In a preclinical model, Glycyrrhizin treatment inhibited HMGB1 expression and release after TBI as evident by the presence of scarce HMGB1 in several nuclei which was completely lost in the injured area of TBI rats. Moreover, it inhibited M1 phenotype and induce M2 phenotype activation of microglia/macrophages (5 days post-TBI) as evident by the reduction in CD86, iNOS, TNF-α, and IL-1β (M1 phenotype markers and functional cytokine mRNA levels) and up-regulation in the markers and the functional cytokine mRNA levels of the M2 phenotype (CD206, Arg1, Ym1, and IL-10) [58].

Ethyl pyruvate is an aliphatic ester developed from an endogenous metabolite [104] that can counteract HMGB1 and neutralize it, further exhibiting biological effects in several diseases [105,106,107]. Its therapeutic potential against experimental TBI relies mainly on HMGB1 inhibition. In a modified Feeney’s weight drop model of TBI, treatment with ethyl pyruvate was shown to inhibit TBI-induced up-regulation of HMGB1 and TLR4 expression. Moreover, it inhibited NF-κB DNA-binding activity, reduced expression of inflammatory molecules (TNF-α, IL-1β, IL-6) and ameliorated locomotor function as evident by beam walking performance, along with cerebral edema and cortical apoptotic cell death [108]. Immunohistochemical findings and western blot analysis from the TBI model in rodents revealed that Ethyl pyruvate treatment reduced HMGB1, TLR4 and RAGE expression after TBI in the pericontusional cerebral tissue. Moreover, the treatment protected BBB permeability as evidenced by increased Occludin, Claudin-5, and ZO-1 levels of BBB tight junction binding proteins, increased total antioxidant status, decreased total oxidant status and oxidative stress index [60].

Omega-3 polyunsaturated fatty acid (ω-3 PUFA) possess a neuroprotective effect which is attributed to the modulation of inflammatory pathways [109]. Supplements of ω-3 PUFA have exerted beneficial effects against clinical [110] and experimental model of TBI [111]. Recently, ω-3 PUFA were shown to exert neuroprotective effects against experimental TBI mainly through the modulation of HMGB1 pathway. In an experimental Feeney DM TBI model, ω-3 PUFA supplementation inhibited TBI-induced activation of microglia by fostering a change from the M1 to the M2 phenotype and decreased the inflammatory response (as evident by down-regulation of TNF-α, IL-1β, IL-6, and IFN-γ). It also reduced neuronal apoptosis post-TBI and enabled neuronal recovery mainly by inhibiting HMGB1 release and translocation as well as HMGB1-mediated stimulation of the TLR4/NF-κB signaling axis [44,111].

Altogether these studies indicate that HMGB1 inhibition by the anti-HMGB1 mAb, Glycyrrhizin, Ethyl pyruvate and ω-3 PUFA exhibit promising effects in experimental TBI mainly by inhibiting the secondary damage. However, the safety profile of these HMGB1-targeted therapies needs to be further explored since mAb treatment has been associated with immunotoxicity and difficult penetration of BBB [112], thus limiting its translational implication. The studies of HMGB1 targeting therapies in TBI are summarized in Table 3.

### 9.2. HMGB1-Targeted Therapy Attenuates EBI Post-SAH

Of importance, EBI after SAH leads to neuroinflammation, brain edema, increased permeability of BBB, apoptosis, and neuronal degeneration [115]. This indicates that attenuating EBI induced by SAH might ameliorate neurological damage and improve the outcome. Also, several pre-clinical findings have reported that inhibition of neuroinflammation exerted significant protection against EBI after SAH [83,116]. Hence, therapeutic inhibition of post-SAH inflammatory cascades might prevent EBI.

Exploring and developing therapeutic agents that reduce EBI and delay cerebral vasospasm (CVS) post-SAH have emerged as effective therapeutic strategies. The anti-HMGB1 mAb attenuated the progression of delayed cerebral vasospasm (CVS) mainly by inhibiting HMGB1 translocation in the VSMCs and reducing SAH up-regulated plasma HMGB1 levels. Also, it suppressed the up-regulation of vasoconstriction-mediating molecules (PAR1, TXA2, AT1, ET_A_). Moreover, the anti-HMGB1 mAb treatment reduced SAH-induced up-regulation of inflammatory molecules (TLR4, IL-6, TNF-α, and iNOS) and ameliorated the neurological symptoms [13], indicating that anti-HMGB1 mAb interrupts the CVS and brain injury after SAH.

In a preclinical model, Glycyrrhizin was shown to suppress the inflammatory response post-SAH mainly by inhibiting the expression of HMGB1. Other mechanisms underlying Glycyrrhizin treatment include reduction of SAH-induced neuronal apoptosis, reduction of inflammatory cytokines (TNF-α and IL-1β), inhibition of BBB permeability post-SAH and reduction of SAH-induced neuronal degeneration [85]. These findings indicate that Glycyrrhizin protects the brain injury of SAH and may present a potential therapy against HMGB1-induced brain inflammation and injury.

In a double-hemorrhage model of SAH, Glycyrrhizic acid ameliorated neurological outcome, inhibited CVS as evident by the increased diameter of BA, and reduced the thickness of the vascular wall. It reduced HMGB1 expression in the BA, and down-regulated the expression of pro-inflammatory cytokines (IL-1β, IL-6, and TNF-α) [87], further suggesting that Glycyrrhizic acid exerted neuroprotection on CVS post-SAH, mainly through inhibition of HMGB1 expression and release, and reduction of pro-inflammatory cytokines.

Several other molecules also demonstrated beneficial effects in experimental SAH, including Resveratrol, Purpurogallin, Melatonin, Rhinacanthin-C, and AG490 (inhibitor of JAK/STAT3). These agents mainly inhibit HMGB1 expression and release, as well as its nuclear to cytosolic translocation, further ameliorating neural apoptosis, brain edema and down-regulating SAH-induced inflammatory markers (TLR4, MyD88, and NF-κB, TNF-α, IL-6, and IL-1β) [76,116,117,118,119] (Table 4). It is, therefore, suggested that HMGB1-targeted therapy might regulate a complex series of inflammatory responses contributing to EBI post-SAH, mainly through suppression of the TLR4/NF-κB signaling cascade.

## 10. Discussion and Future Suggestions

Current research directions in TBI are mainly focused on preventing the secondary damage and on inhibiting the neuroinflammation that contributes to both acute and chronic stages. HMGB1 has been suggested to participate in the progression of TBI and might represent a promising target. During TBI, cellular damage/death might cause HMGB1 to translocate from the nucleus to extracellular space resulting in microglial activation and leading to further release of HMGB1 [103]. Elucidation of HMGB1 translocation and release process along with HMGB1-driven activation of the TLR4/NF-κB signaling pathway post-TBI may prove beneficial in understanding the mechanism of TBI-induced secondary brain damage [111].

HMGB1-based agents (anti-HMGB1 mAb and Glycyrrhizin) have been shown to exert neuroprotective effects in experimental TBI models including amelioration of neurological outcome, inhibition of translocation of HMGB1, down-regulation of TBI-induced neuroinflammatory responses, prevention of BBB breakdown and reduction of microglial activation [12,59]. Similarly, HMGB1 neutralization inhibited SAH-induced EBI mainly by protecting BBB permeability, blocking HMGB1 expression, release, and translocation, and down-regulating SAH-induced neuroinflammatory response. These findings suggest that HMGB1 might represent an extracellular target against several forms of brain injury such as TBI and EBI induced by SAH. Nevertheless, when evaluating the beneficial therapeutic effect of HMGB1, it will be important to assess its effect on long-term cognitive function. The prolonged absence of HMGB1 might impair the cognitive function despite its beneficial effects on preventing secondary injury [61]. Moreover, assessment of the duration of beneficial effects upon HMGB1 neutralization/inhibition must be determined [12,102].

Despite the compelling findings from experimental studies, no clinical data reporting beneficial effects of HMGB1-targeted therapies against TBI and SAH-induced EBI are available. Moreover, extensive investigation of HMGB1 targeting agents against brain injuries is warranted since the degree, the location, and the duration of HMGB1 neutralization might be complex and variable due to different insult etiologies [61]. HMGB1 has therefore emerged as an attractive therapeutic target against several HMGB1-mediated inflammatory diseases [23,122]. A future challenge for the clinical translation of HMGB1-based therapies is the development of isoform-specific HMGB1 inhibitors that could suppress the damage without inhibiting tissue regeneration because the thiol-HMGB1 isoform may possess a tissue protecting role in inflammation, injury, and regeneration. All currently available HMGB1 antagonists bind to all the three redox forms of HMGB1 [23] and therefore future studies should be focused on blocking the harmful disulfide-HMGB1 isoform that exerts inflammatory role [23,25].

## 11. Conclusions

Altogether HMGB1 protein is a key mediator of neuroinflammatory response and contributes to the pathogenesis of TBI as well as to the secondary damage post-TBI and in the EBI following SAH. Moreover, HMGB1 can be used as a biomarker to predict functional outcome after TBI and EBI after SAH while its therapeutic neutralization may prove beneficial in inhibiting the secondary damage following these injuries.

## Figures and Tables

**Table 1:** Summaries of findings reporting biomarker potential of HMGB1 to predict outcome after TBI.

S.N.	Study Details	HMGB1 Levels	Observations	References
**1**	CSF obtained from adult TBI patients (*n* = 26) with GCS (3T-12T) and normal pressure hydrocephalus (NPH) patients as controls (*n* = 9)	Increased	Up-regulated expression of HMGB1 (CSF) was observed in TBI patients with extra ventricular drainage for increased ICP, where the highest HMGB1 expression was observed over the first 72 h.	[45]
**2**	Observational clinical study involving TBI patients (*n* = 106) and healthy controls (*n* = 106)	Increased	HMGB1 expression in plasma was elevated in TBI patients compared to healthy controls. Plasma HMGB1 levels were suggested as an independent predictor for 1-yr mortality and unfavorable outcome of patients as determine by multivariate analysis.	[66]
**3**	Ventricular CSF was obtained from pediatric TBI (*n* = 27) and normal control (*n* = 12)	Increased	Peak HMGB1 levels were inversely and independently correlated with the favorable GOS scores at 6 months after TBI. Temporal profiles of HMGB1 levels were reported to be 1.78 ± 0.29 (control group), 5.73 ± 1.45 (0–24 h), 5.16 ± 1.73 (25–48 h), 4.13 ± 0.75 (49–72 h) and 3.80 ± 0.90 (>72 h) after TBI.	[67]
**4**	Human postmortem samples from TBI patients (*n* = 25)	Increased	There was a nucleo-cytoplasmic translocation of HMGB1. HMGB1 was mainly localized in the cytoplasm of phagocytic microglia in the contused area between 2–20 days post-TBI.	[19]

TBI Traumatic brain injury; HMGB1, High mobility group box 1; CSF, Cerebrospinal fluid; GCS, Glasgow coma scale; GOS, Glasgow outcome scale.

**Table 2:** Studies indicating the biomarker potential of HMGB1 after SAH.

S.N.	Study Type	Study Design	HMGB1 Levels	Observations	References
**1**	Clinical investigation with aneurysmal SAH patients (*n* = 53)	Retrospective observational study	Serum	Serum HMGB1 levels were elevated in SAH patients from day 1 and remained elevated until day 13 in patients developing cerebral vasospasm reflecting a biomarker potential.	[90]
**2**	A human study with aneurysmal SAH and controls (*n* = 40)	CSF was collected 7 days post-SAH and functional outcome was assessed using GOS.	CSF	Elevated CSF HMGB1 levels were independently correlated with the unfavorable outcome at three months after SAH.	[91]
**3**	Clinical study with aneurysmal SAH patients (*n* = 149) and healthy controls (*n* = 50)	Prospective, 2-center study evaluating C/G HMGB1 SNP rs2249825.	Protein	The presence of minor allele G of rs2249825 was correlated with a higher risk of DCI, or cerebral infarction after aneurysmal SAH reflecting an up-regulated expression of HMGB1 protein.	[92]
**4**	Clinical investigation with aneurysmal SAH patients (*n* = 47)	Prospective population-based study	Plasma	Plasma concentration of HMGB1 detected during the early 24 h was not correlated with the neurological outcome. In addition, as suggested by the linear regression analysis no correlation between HMGB1 levels and neurological outcome was observed during the follow up (first 5 days).	[89]
**5**	A clinical study with aneurysmal SAH patients (*n* = 10) and healthy controls (*n* = 8)	The subjects were followed until death (endpoint) or 3 months post-SAH. The primary outcome was the functional state as determined by the GOS score and secondary outcome was mortality (in-hospital).	CSF	HMGB1 (CSF) levels were up-regulated in the SAH patients when compared to normal controls where initial levels and gradual changes in HMGB1 levels (CSF) were associated with neurological outcomes.	[93]
**6**	A clinical study with aneurysmal SAH patients (*n* = 347) and healthy controls (*n* = 150)	The study endpoints were mortality after 1 year, mortality in-hospital, cerebrovasospasm and poor functional outcome following 1 year.	Plasma	Plasma levels of HMGB1 were elevated in SAH patients as compared to healthy controls. HMGB1 levels (plasma) were correlated with the poor functional outcome and mortality after 1 year, in-hospital mortality and cerebrovasospasm as determined by multivariate analysis.	[88]
**7**	A clinical study with SAH patients (*n* = 9) and healthy controls (*n* = 7)		CSF	Compared to SAH patients, HMGB1 expression was below the level of detection in the CSF of control subjects reflecting that HMGB1 release might be specific to brain injury. HMGB1 levels (CSF) were retrospectively associated with the neurological outcome, as determined by the Hunt and Hess grading scale.	[94]
**8**	Human studies with SAH populations (*n* = 39) and controls (13)	Samples (CSF) were taken on days 3, 7, and 14 after admission.	CSF	HMGB1 (CSF) levels were elevated in patients with unfavorable outcomes after SAH, reflecting a role in brain damage post-SAH.	[95]

SAH, Subarachnoid hemorrhage; HMGB1, High mobility group box-1; CSF, Cerebrospinal fluid; GOS, Glasgow outcome scale; SNP, Single-nucleotide-polymorphism.

**Table 3:** Studies targeting HMGB1 in TBI.

S.N.	Study Model	Intervention and Dosing Schedule	Observations	References
**1**	FPI-induced TBI in adult male Wistar rats	Anti-HMGB1 mAb (1 mg/kg, I.V.) was administered at 5 min and 6 h after TBI	Anti-HMGB1 mAb prevented neuronal death, attenuated accretion of activated microglia in the rat cortex, inhibited translocation of HMGB1 and ameliorated motor function. Treatment with anti-HMGB1 mAb exerted beneficial effects on motor and cognitive function (only for 2 weeks after TBI).	[102]
**2**	ICH-induced brain injury in male Wistar rats	Anti-HMGB1 mAb (1 mg/kg, I.V.) was administered immediately and 6 h after ICH.	Anti-HMGB1 mAb inhibited the HMGB1 release into the extracellular space, decreased serum HMGB1 levels and reduced brain edema by protecting BBB integrity, reduced activated microglia and decreased the expression of inflammation-related factors (TNF-α, iNOS, IL-1β, IL-6, IL-8R, COX-2, MMP2, MMP9 and VEGF 121). Anti-HMGB1 mAb administration suppressed oxidative stress and ameliorated behavioral performance.	[103]
**3**	FPI-induced TBI in adult male Wistar rats	Anti-HMGB1 mAb (1 mg/kg, I.V.) was administered at 5 min and 6 h after TBI	Anti-HMGB1 mAb reduced FPI-induced brain edema, inhibited translocation of HMGB1, protected BBB integrity, suppressed expression of inflammatory molecules (TNF-α, iNOS, HIF-1α, COX-2, VEGF-A189, and VEGF-A165) and improved motor function.	[12]
**4**	CCI-induced TBI in male C57Bl/6 mice	GL (for acute recovery study) (50 mg/kg, I.P.) was administered 1 h, 6 h, 1 d and 2 d post-injury, plus 1 h pre-injury. GL (for chronic recovery study) was administered 1 h pre-injury, at 1 h, 6 h post-injury, plus once daily for 7 added days for 1 week.	GL reduced brain HMGB1 levels and brain edema at an acute time point of 3 days post-injury (acute outcomes upon HMGB1 neutralization). Treatment with GL ameliorated short-term spatial memory and motor learning impairments as well as reduced an elevation in hippocampal microglial reactivity (chronic outcomes on HMGB1 inhibition).	[21]
**5**	TBI induced by modified Feeney’s free weight drop method in male SD rats	GL (10 mg/kg, I.V.) administered 30 min after TBI	GL administration reduced overexpression of HMGB1, TLR4, and RAGE, NF-κB DNA-binding activity and inhibited expression of inflammatory cytokines (TNF-α, IL-1β, and IL-6). GL treatment decreased brain edema and ameliorated the motor function as evident by beam walking test.	[64]
**6**	FPI-induced TBI in adult male Wistar rats	GL (0.25, 1.0 or 4.0 mg/kg, I.V.) was administered 5 min after injury	GL inhibited the translocation of HMGB1 in neurons at the injured area, protected the BBB permeability and ameliorated motor functions. GL administration inhibited TBI-induced up-regulation of inflammatory molecules (TNF-α, IL-1β, and IL-6) post-TBI.	[59]
**7**	ICH-induced injury in male SD rats	GL (50 mg/kg) was administered 20 min post-ICH, and then once daily for 3 days.	GL reduced ICH-induced increase of the brain water content, ameliorated neurological deficit induced by ICH. Treatment with GL ameliorated ICH-induced neuron loss inside hematoma as evident by an increased number of NeuN-positive cells.	[113]
**8**	DAI-induced brain injury in adult SD rats	Glycyrrhizic acid (GA) (10 mg/kg, I.V.) administered 30 min before DAI	Pre-treatment with GA ameliorated motor and cognitive deficits, inhibited DAI-induced extracellular expression of HMGB1, reduced neuronal apoptosis, protected BBB integrity and inhibited expression of pro-inflammatory cytokines (TNF-α, MMP-9, and IL-6).	[114]
**9**	Weight-dropping TBI model in male adult SD rats	Ethyl pyruvate (EP) (75 mg/kg, I.P.) prepared at 30 min, 1.5 h, and 6 h	EP treatment decreased the post-traumatic up-regulation in HMGB-1, TLR4 and RAGE expressions, reduced brain edema, increased BBB permeability as evident by increased expression of occludin, claudin-5 and ZO-1 expression (tight junction proteins of BBB). EP suppressed proapoptotic bax and active caspase 3 expression, increased anti-apoptotic bcl-2 levels, decreased total oxidant status and oxidative stress and increased total antioxidant status post-TBI.	[60]
**10**	Feeney’s weight drop model in male SD rats	EP (75 mg/kg, I.P.) administered 5 min, 1 h, and 6 h post-TBI	EP treatment ameliorated performance in beam walking, brain edema, and cortical apoptotic cell death. EP treatment inhibited expression of HMGB1 and TLR4, NF-κB DNA-binding activity and down-regulated expression of inflammatory mediators (IL-1β, TNF-α, and IL-6).	[108]
**11**	CCI-induced TBI in male SD rats	Minocycline (90 mg/kg, I.P.) was administered 10 min and 20 h after injury	Minocycline treatment attenuated nuclear to cytosolic translocation of HMGB1, reduced activation of microglia (in the ipsilateral cortex, hippocampus, and thalamus), inhibited neurodegeneration (FJB-positive neurons) and delayed motor recovery and improved spatial memory acquisition as evident by MWM test.	[65]
**12**	Feeney DM TBI model in adult male SD rats	ω-3 PUFA (2 mL/kg, I.P.) was administered 30 min after TBI, each day for 1 week.	Treatment with ω-3 PUFA demonstrated neuroprotection against TBI by manipulating microglial polarization via SIRT1-mediated deacetylation of the HMGB1-NF-κB signaling axis. ω-3 PUFA suppressed nucleocytoplasmic translocation of HMGB1, down-regulated acetylation of HMGB1, reduced TBI-induced expression of inflammatory mediators (HMGB1, TNF-α, IL-1β, IL-6), and protected TBI-induced neuronal apoptosis.	[44]
**13**	Feeney DM TBI model in adult male SD rats	ω-3 PUFA (2 mL/kg, I.P.) was administered 30 min post-TBI, each day for 1 week.	ω-3 PUFA supplementation inhibited HMGB1 nuclear translocation, reduced the secretion and expression of HMGB1 in neurons and microglia in the lesioned areas. ω-3 PUFA supplementation reduced TBI-mediated activation of microglia and expression of inflammatory mediators (TNF-α, IL-1β, IL-6, and IFN-γ), lowered brain edema, reduced neuronal apoptosis, and ameliorated neurological functions post-TBI.	[111]

TBI, Traumatic brain injury, HMGB1, High mobility group box-1; ω-3 PUFA, Omega-3 polyunsaturated fatty acid; FPI, Fluid percussion injury, Anti-HMGB1 mAb, Anti-HMGB1 monoclonal antibody; SD, Sprague-Dawley; TNF-α, Tumor necrosis factor-alpha; NF-κB, Nuclear factor κ-light chain enhancer of activated B cells; IL, Interleukin; IFN-γ, Interferon-gamma; I.P., Intraperitoneal; I.V., Intravenous; BBB, Blood–brain barrier; VEGF, Vascular endothelial growth factor, HIF-1α, Hypoxia-inducible factor-1 α; COX-2, Cyclooxygenase-2; iNOS, Inducible nitric oxide synthase; CCI, Controlled cortical impact; ICH, Intracerebral hemorrhage; DAI, Diffuse axonal injury; MMP, Matrix metalloproteinase; EP, Ethyl Pyruvate; GL, Glycyrrhizin; SIRT, Sirtuin; MWM, Morris water maze.

**Table 4:** Summaries of studies targeting HMGB1 against EBI induced by SAH.

S.N.	Study Model	Intervention and Dosing Schedule	Observations	References
**1**	Endovascular puncture model of SAH adult male Wistar rats	Anti-HMGB1 mAb (1 mg/kg, I.V.) was administered post-SAH, twice at an interval of 24 h.	Anti-HMGB1 mAb suppressed nuclear translocation of HMGB1, suppressed up-regulation of inflammation-related factors (TLR4, IL-6, TNF-α, and iNOS) and inhibited vasoconstriction-mediating receptors (PAR1, TXA2, AT1, ET_A_). Anti-HMGB1 mAb administration ameliorated neurological symptoms and body weight post-SAH.	[13]
**2**	Endovascular perforation model of SAH adult male SD rats	Anti-HMGB1 mAb (1 mg/kg, I.V.) was administered post-SAH, twice at an interval of 24 h.	Anti-HMGB1 mAb attenuated microglial activation, brain edema, and ameliorated neurological dysfunction. Anti-HMGB1 mAb reversed the elevated expression of HMGB1 in the cortex after SAH and reversed VSMC phenotypic switching and vascular remodeling.	[81]
**3**	Prechiasmatic cistern SAH model in male SD rats	Glycyrrhizin (GL) (15 mg/kg, I.P.) was administered immediately and then 6, 12 and 18 h post-SAH.	Treatment with GL decreased HMGB1-positive cells, down-regulated mRNA and protein levels of HMGB1, preserved BBB permeability and attenuated neuronal cell death and apoptosis after SAH. GL treatment suppressed the SAH-induced up-regulation of inflammatory molecules (TNF-α and IL-1β) and significantly improved neurological scores.	[85]
**4**	SAH in male SD rats	GL (5 mg/kg/day) was administered 24 h prior (precondition) and 1 h post-SAH (treatment).	GL administration demonstrated anti-inflammatory effects in SAH-induced vasospasms. Treatment with GL elevated the expression of PPAR-γ protein and mRNA (pre-conditioning) and PPAR-δ mRNA (treatment and preconditioning).	[120]
**5**	Modified double-hemorrhage SAH model in male SD rats	Glycyrrhizic acid (GA) (10 mg/kg, I.P.) was administered immediately after SAH and was continued for three consecutive days.	Administration of GA improved neurological function post-SAH reduced SAH-induced increased expression of HMGB1 protein (in a basilar artery) and inflammatory mediators (TNF-α, IL-6, and IL-1β).	[87]
**6**	Endovascular perforation induced SAH in male SD rats	AG490 (inhibitor of JAK2/STAT3) (2 mL, I.V.) was administered 30 min before SAH	Treatment with AG490 after SAH significantly down-regulated JAK2/STAT3 phosphorylation, inhibited HMGB1 expression and its translocation, decreased cortical apoptosis, brain edema and ameliorated neurological deficits.	[76]
**7**	Prechiasmatic cistern SAH model in male SD rats	Resveratrol (60 mg/kg, I.P.) was administered at 2 and 12 h post-SAH.	Treatment with Resveratrol exerted neuroprotection by inhibiting an up-regulated expression of HMGB1, TLR4, MyD88, and NF-κB post-SAH, ameliorating neural apoptosis, brain edema, and impairments in neurological behavior impairment.	[116]
**8**	Double-hemorrhage SAH model in male SD rats	Purpurogallin (100, 200 and 600 mg/kg/day) was administered 1 h after SAH.	Purpurogallin demonstrated its neuroprotective effects by inhibiting IL-6 and TNF-α mRNA expression and decreasing HMGB1 expression (protein and mRNA). Purpurogallin also exerted SAH-induced vasoconstriction (dose-dependent), ameliorated neurological deficit as evident by motor deficit index.	[117]
**9**	Prechiasmatic cistern SAH model in SD rats	Melatonin (150 mg/kg, I.P.) was administered 2 and 24 h after SAH.	Melatonin exerted neuroprotective effects against SAH and attenuated neurofunctional dysfunction post-SAH mainly by inhibiting expression of HMGB1, TLR4, NF-κB, MyD88, IL-1β, TNF-α, IL-6, and iNOS. Melatonin treatment also ameliorated spatial learning and memory deficit as evident by the MWM test.	[119]
**10**	Rodents SAH model in male SD rats	Rhinacanthin-C (RCT-C) (100, 200, and 400 µmol/kg/day) was administered orally 1 h after SAH and every 12 h.	RCT-C treatment exerted neuroprotection by inhibiting the expression of HMGB1 (protein and mRNA), down-regulating the expression of inflammatory mediators (IL-1β, TNF-α, IL-6,) attenuating brain apoptosis post-SAH (reduced caspase-3- and caspase-9a). RCT-C attenuated SAH-induced vasoconstriction, reduced GFAP+ microglia and increased NeuN+ neurons compared to SAH animals.	[118]
**11**	Double-hemorrhage SAH model in male SD rats	4OGOMV (100, 200 and 400 µg/kg/day) was administered 1 h post-SAH.	4. OGOMV exerted neuroprotective against SAH by attenuating SAH-induced vasoconstriction, ameliorating neurological deficit as evident by improved MDI, inhibiting expression of HMGB1 protein and pro-inflammatory mediators (IL-1β, IL-6, IL-8, and MCP-1).	[121]

SAH, Subarachnoid hemorrhage; HMGB1, high mobility group box-1; TLR4, Toll-like receptor-4; Anti-HMGB1 mAb, Anti-HMGB1 monoclonal antibody; SD, Sprague-Dawley; VSMC, vascular smooth muscle cell; I.P., Intraperitoneal; I.V., Intravenous; BBB, Blood–brain barrier; iNOS, Inducible nitric oxide synthase; eNOS, endothelial nitric oxide synthase; TNF-α, Tumor necrosis factor-alpha; IL, Interleukin; NF-κB, Nuclear factor κ-light chain enhancer of activated B cells; MyD88, Myeloid differentiation factor 88; TXA2, Thromboxane A2; PAR1, Protease-activated receptor-1; AT1, Angiotensin II type 1; ET_A_, Endothelin type A; PPAR, Peroxisome proliferator-activated receptor-γ; GL, Glycyrrhizin; GA, Glycyrrhizic acid; MWM, Morris water maze; 4OGOMV, 4′-O-β-*D*-glucosyl-5-O-methylvisamminol; MDI, Motor deficit index; MCP-1, Monocyte chemoattractant protein-1; JAK2, Janus kinase 2; STAT3, signal transducer and activator of transcription 3.

## Abbreviations

TBI	Traumatic brain injury;
EBI	Early brain injury;
SAH	Subarachnoid hemorrhage;
HMGB1	High mobility group box 1;
RAGE	Receptor for advanced glycation end products;
TLRs	Toll-like receptors;
DAMP	Damage-associated molecular patterns;
JAK	Janus kinases;
STAT3	Signal transducer and activator of transcription 3;
MD-2	Myeloid differentiation factor-2;
BBB	Blood–brain barrier;
CNS	Central nervous system;
GFAP	Glial fibrillary acidic protein;
IL	Interleukin;
IBA1	Ionized calcium-binding adapter molecule 1;
iNOS	Inducible nitric oxide synthase;
NADPH	Nicotinamide-adenine dinucleotide phosphate;
NF-κB	Nuclear factor κ light chain enhancer of activated B cells;
TNF-α	Tumor necrosis factor-α;
IR	Immunoreactivity;
FPI	Fluid percussion injury;
Anti-HMGB1 mAb	Anti-HMGB1 monoclonal antibody;
SD	Sprague-Dawley;
IFN-γ	Interferon-gamma;
VEGF	Vascular endothelial growth factor;
CCI	Controlled cortical impact;
ICP	Intracranial pressure;
ICH	Intracerebral hemorrhage;
DAI	Diffuse axonal injury;
WT	Wild-type;
MMP	Matrix metalloproteinase;
MAPK	Mitogen-activated protein kinase
MAP2	Microtubule-associated protein 2;
AQP4	Aquaporin-4;
VSMC	Vascular smooth muscle cells;
ICAM	Intercellular adhesion molecule;
VCAM	Vascular cell-adhesion molecule;
PI3K/AKT	Phosphoinositide 3-kinases/protein kinase B;
GCS	Glasgow Coma Scale

## References

[1] Saatman K.E., Duhaime A.-C., Bullock R., Maas A.I., Valadka A., Manley G.T. (2008). Classification of traumatic brain injury for targeted therapies. J. Neurotrauma.

[2] McIntosh T.K., Smith D.H., Meaney D.F., Kotapka M.J., Gennarelli T.A., Graham D.I. (1996). Neuropathological sequelae of traumatic brain injury: Relationship to neurochemical and biomechanical mechanisms. Lab. Investig. A J. Tech. Methods Pathol..

[3] Bragge P., Synnot A., Maas A.I., Menon D.K., Cooper D.J., Rosenfeld J.V., Gruen R.L. (2016). A state-of-the-science overview of randomized controlled trials evaluating acute management of moderate-to-severe traumatic brain injury. J. Neurotrauma.

[4] Hawryluk G.W., Bullock M.R. (2016). Past, present, and future of traumatic brain injury research. Neurosurg. Clin..

[5] McKee A.C., Daneshvar D.H. (2015). The neuropathology of traumatic brain injury. Handbook of Clinical Neurology.

[6] Werner C., Engelhard K. (2007). Pathophysiology of traumatic brain injury. BJA Br. J. Anaesth..

[7] Sulhan S., Lyon K.A., Shapiro L.A., Huang J.H. (2020). Neuroinflammation and blood–brain barrier disruption following traumatic brain injury: Pathophysiology and potential therapeutic targets. J. Neurosci. Res..

[8] Henry R.J., Doran S.J., Barrett J.P., Meadows V.E., Sabirzhanov B., Stoica B.A., Loane D.J., Faden A.I. (2019). Inhibition of miR-155 limits neuroinflammation and improves functional recovery after experimental traumatic brain injury in mice. Neurotherapeutics.

[9] Long X., Yao X., Jiang Q., Yang Y., He X., Tian W., Zhao K., Zhang H. (2020). Astrocyte-derived exosomes enriched with miR-873a-5p inhibit neuroinflammation via microglia phenotype modulation after traumatic brain injury. J. Neuroinflamm..

[10] Sims G.P., Rowe D.C., Rietdijk S.T., Herbst R., Coyle A.J. (2009). HMGB1 and RAGE in inflammation and cancer. Annu. Rev. Immunol..

[11] Aucott H., Lundberg J., Salo H., Klevenvall L., Damberg P., Ottosson L., Andersson U., Holmin S., Harris H.E. (2018). Neuroinflammation in response to intracerebral injections of different HMGB1 redox isoforms. J. Innate Immun..

[12] Okuma Y., Liu K., Wake H., Zhang J., Maruo T., Date I., Yoshino T., Ohtsuka A., Otani N., Tomura S. (2012). Anti–high mobility group box-1 antibody therapy for traumatic brain injury. Ann. Neurol..

[13] Haruma J., Teshigawara K., Hishikawa T., Wang D., Liu K., Wake H., Mori S., Takahashi H.K., Sugiu K., Date I. (2016). Anti-high mobility group box-1 (HMGB1) antibody attenuates delayed cerebral vasospasm and brain injury after subarachnoid hemorrhage in rats. Sci. Rep..

[14] Fu L., Liu K., Wake H., Teshigawara K., Yoshino T., Takahashi H., Mori S., Nishibori M. (2017). Therapeutic effects of anti-HMGB1 monoclonal antibody on pilocarpine-induced status epilepticus in mice. Sci. Rep..

[15] Fujita K., Motoki K., Tagawa K., Chen X., Hama H., Nakajima K., Homma H., Tamura T., Watanabe H., Katsuno M. (2016). HMGB1, a pathogenic molecule that induces neurite degeneration via TLR4-MARCKS, is a potential therapeutic target for Alzheimer’s disease. Sci. Rep..

[16] Coco D.L., Veglianese P., Allievi E., Bendotti C. (2007). Distribution and cellular localization of high mobility group box protein 1 (HMGB1) in the spinal cord of a transgenic mouse model of ALS. Neurosci. Lett..

[17] Paudel Y.N., Angelopoulou E., Piperi C., Othman I., Shaikh M.F. (2020). Implication of HMGB1 signaling pathways in Amyotrophic lateral sclerosis (ALS): From molecular mechanisms to pre-clinical results. Pharmacol. Res..

[18] Sasaki T., Liu K., Agari T., Yasuhara T., Morimoto J., Okazaki M., Takeuchi H., Toyoshima A., Sasada S., Shinko A. (2016). Anti-high mobility group box 1 antibody exerts neuroprotection in a rat model of Parkinson’s disease. Exp. Neurol..

[19] Gao T.-L., Yuan X.-T., Yang D., Dai H.-L., Wang W.-J., Peng X., Shao H.-J., Jin Z.-F., Fu Z.-J. (2012). Expression of HMGB1 and RAGE in rat and human brains after traumatic brain injury. J. Trauma Acute Care Surg..

[20] Chen X., Zhang J., Kim B., Jaitpal S., Meng S.S., Adjepong K., Imamura S., Wake H., Nishibori M., Stopa E.G. (2019). High-mobility group box-1 translocation and release after hypoxic ischemic brain injury in neonatal rats. Exp. Neurol..

[21] Webster K.M., Shultz S.R., Ozturk E., Dill L.K., Sun M., Casillas-Espinosa P., Jones N.C., Crack P.J., O’Brien T.J., Semple B.D. (2019). Targeting high-mobility group box protein 1 (HMGB1) in pediatric traumatic brain injury: Chronic neuroinflammatory, behavioral, and epileptogenic consequences. Exp. Neurol..

[22] Webster K.M., Sun M., Crack P.J., O’Brien T.J., Shultz S.R., Semple B.D. (2019). Age-dependent release of high-mobility group box protein-1 and cellular neuroinflammation after traumatic brain injury in mice. J. Comp. Neurol..

[23] Andersson U., Yang H., Harris H. (2018). Extracellular HMGB1 as a therapeutic target in inflammatory diseases. Expert Opin. Ther. Targets.

[24] Paudel Y.N., Angelopoulou E., Piperi C., Balasubramaniam V.R., Othman I., Shaikh M.F. (2019). Enlightening the role of high mobility group box 1 (HMGB1) in inflammation: Updates on receptor signalling. Eur. J. Pharmacol..

[25] Andersson U., Yang H., Harris H. (2018). High-mobility group box 1 protein (HMGB1) operates as an alarmin outside as well as inside cells. Semin. Immunol..

[26] O’Connor K.A., Hansen M.K., Pugh C.R., Deak M.M., Biedenkapp J.C., Milligan E.D., Johnson J.D., Wang H., Maier S.F., Tracey K.J. (2003). Further characterization of high mobility group box 1 (HMGB1) as a proinflammatory cytokine: Central nervous system effects. Cytokine.

[27] Hori O., Brett J., Slattery T., Cao R., Zhang J., Chen J.X., Nagashima M., Lundh E.R., Vijay S., Nitecki D. (1995). The receptor for advanced glycation end products (RAGE) is a cellular binding site for amphoterin mediation of neurite outgrowth and co-expression of rage and amphoterin in the developing nervous system. J. Biol. Chem..

[28] Yang H., Lundbäck P., Ottosson L., Erlandsson-Harris H., Venereau E., Bianchi M.E., Al-Abed Y., Andersson U., Tracey K.J., Antoine D.J. (2012). Redox modification of cysteine residues regulates the cytokine activity of high mobility group box-1 (HMGB1). Mol. Med..

[29] Yang H., Wang H., Ju Z., Ragab A.A., Lundbäck P., Long W., Valdes-Ferrer S.I., He M., Pribis J.P., Li J. (2015). MD-2 is required for disulfide HMGB1–dependent TLR4 signaling. J. Exp. Med..

[30] Palmblad K., Schierbeck H., Sundberg E., Horne A.-C., Harris H.E., Henter J.-I., Antoine D.J., Andersson U. (2014). High systemic levels of the cytokine-inducing HMGB1 isoform secreted in severe macrophage activation syndrome. Mol. Med..

[31] Venereau E., Casalgrandi M., Schiraldi M., Antoine D.J., Cattaneo A., De Marchis F., Liu J., Antonelli A., Preti A., Raeli L. (2012). Mutually exclusive redox forms of HMGB1 promote cell recruitment or proinflammatory cytokine release. J. Exp. Med..

[32] Dinet V., Petry K.G., Badaut J. (2019). Brain-immune interactions and neuroinflammation after traumatic brain injury. Front. Neurosci..

[33] Maas A.I., Stocchetti N., Bullock R. (2008). Moderate and severe traumatic brain injury in adults. Lancet Neurol..

[34] Maas A.I., Menon D.K., Adelson P.D., Andelic N., Bell M.J., Belli A., Bragge P., Brazinova A., Büki A., Chesnut R.M. (2017). Traumatic brain injury: Integrated approaches to improve prevention, clinical care, and research. Lancet Neurol..

[35] Morganti-Kossmann M.C., Semple B.D., Hellewell S.C., Bye N., Ziebell J.M. (2019). The complexity of neuroinflammation consequent to traumatic brain injury: From research evidence to potential treatments. Acta Neuropathol..

[36] Loane D.J., Faden A.I. (2010). Neuroprotection for traumatic brain injury: Translational challenges and emerging therapeutic strategies. Trends Pharmacol. Sci..

[37] Barrett J.P., Henry R.J., Shirey K.A., Doran S.J., Makarevich O.D., Ritzel R.M., Meadows V.A., Vogel S.N., Faden A.I., Stoica B.A. (2020). Interferon-β plays a detrimental role in experimental traumatic brain injury by enhancing neuroinflammation that drives chronic neurodegeneration. J. Neurosci..

[38] Kumar A., Loane D.J. (2012). Neuroinflammation after traumatic brain injury: Opportunities for therapeutic intervention. Brain Behav. Immun..

[39] Tang D., Billiar T.R., Lotze M.T. (2012). A Janus tale of two active high mobility group box 1 (HMGB1) redox states. Mol. Med..

[40] Schiraldi M., Raucci A., Muñoz L.M., Livoti E., Celona B., Venereau E., Apuzzo T., De Marchis F., Pedotti M., Bachi A. (2012). HMGB1 promotes recruitment of inflammatory cells to damaged tissues by forming a complex with CXCL12 and signaling via CXCR4. J. Exp. Med..

[41] Terrando N., Yang T., Wang X., Fang J., Cao M., Andersson U., Erlandsson H.H., Ouyang W., Tong J. (2016). Systemic HMGB1 neutralization prevents postoperative neurocognitive dysfunction in aged rats. Front. Immunol..

[42] Lee S., Nam Y., Koo J.Y., Lim D., Park J., Ock J., Kim J., Suk K., Park S.B. (2014). A small molecule binding HMGB1 and HMGB2 inhibits microglia-mediated neuroinflammation. Nat. Chem. Biol..

[43] Takizawa T., Shibata M., Kayama Y., Shimizu T., Toriumi H., Ebine T., Unekawa M., Koh A., Yoshimura A., Suzuki N. (2017). High-mobility group box 1 is an important mediator of microglial activation induced by cortical spreading depression. J. Cereb. Blood Flow Metab..

[44] Chen X., Chen C., Fan S., Wu S., Yang F., Fang Z., Fu H., Li Y. (2018). Omega-3 polyunsaturated fatty acid attenuates the inflammatory response by modulating microglia polarization through SIRT1-mediated deacetylation of the HMGB1/NF-κB pathway following experimental traumatic brain injury. J. Neuroinflamm..

[45] Laird M.D., Shields J.S., Sukumari-Ramesh S., Kimbler D.E., Fessler R.D., Shakir B., Youssef P., Yanasak N., Vender J.R., Dhandapani K.M. (2014). High mobility group box protein-1 promotes cerebral edema after traumatic brain injury via activation of toll-like receptor 4. Glia.

[46] Buchanan M.M., Hutchinson M., Watkins L.R., Yin H. (2010). Toll-like receptor 4 in CNS pathologies. J. Neurochem..

[47] Colonna M., Butovsky O. (2017). Microglia function in the central nervous system during health and neurodegeneration. Annu. Rev. Immunol..

[48] Qi R., Wang X. (2020). Inhibition of miR-429 improves neurological recovery of traumatic brain injury mice and attenuates microglial neuroinflammation. Int. Immunopharmacol..

[49] Paudel Y.N., Angelopoulou E., Bhuvan K., Piperi C., Othman I. (2019). High mobility group box 1 (HMGB1) protein in Multiple Sclerosis (MS): Mechanisms and therapeutic potential. Life Sci..

[50] Takada S., Sakakima H., Matsuyama T., Otsuka S., Nakanishi K., Norimatsu K., Itashiki Y., Tani A., Kikuchi K. (2020). Disruption of Midkine gene reduces traumatic brain injury through the modulation of neuroinflammation. J. Neuroinflamm..

[51] Turtzo L.C., Lescher J., Janes L., Dean D.D., Budde M.D., Frank J.A. (2014). Macrophagic and microglial responses after focal traumatic brain injury in the female rat. J. Neuroinflamm..

[52] Karve I.P., Taylor J.M., Crack P.J. (2016). The contribution of astrocytes and microglia to traumatic brain injury. Br. J. Pharmacol..

[53] Hsieh C.L., Kim C.C., Ryba B.E., Niemi E.C., Bando J.K., Locksley R.M., Liu J., Nakamura M.C., Seaman W.E. (2013). Traumatic brain injury induces macrophage subsets in the brain. Eur. J. Immunol..

[54] Kumar A., Alvarez-Croda D.-M., Stoica B.A., Faden A.I., Loane D.J. (2016). Microglial/macrophage polarization dynamics following traumatic brain injury. J. Neurotrauma.

[55] Kumar A., Barrett J.P., Alvarez-Croda D.-M., Stoica B.A., Faden A.I., Loane D.J. (2016). NOX2 drives M1-like microglial/macrophage activation and neurodegeneration following experimental traumatic brain injury. Brain Behav. Immun..

[56] Bu W., Ren H., Deng Y., Del Mar N., Guley N.M., Moore B.M., Honig M.G., Reiner A. (2016). Mild traumatic brain injury produces neuron loss that can be rescued by modulating microglial activation using a CB2 receptor inverse agonist. Front. Neurosci..

[57] Wang J., Li R., Peng Z., Hu B., Rao X., Li J. (2020). HMGB1 participates in LPS-induced acute lung injury by activating the AIM2 inflammasome in macrophages and inducing polarization of M1 macrophages via TLR2, TLR4, and RAGE/NF-κB signaling pathways. Int. J. Mol. Med..

[58] Gao T., Chen Z., Chen H., Yuan H., Wang Y., Peng X., Wei C., Yang J., Xu C. (2018). Inhibition of HMGB1 mediates neuroprotection of traumatic brain injury by modulating the microglia/macrophage polarization. Biochem. Biophys. Res. Commun..

[59] Okuma Y., Liu K., Wake H., Liu R., Nishimura Y., Hui Z., Teshigawara K., Haruma J., Yamamoto Y., Yamamoto H. (2014). Glycyrrhizin inhibits traumatic brain injury by reducing HMGB1–RAGE interaction. Neuropharmacology.

[60] Evran S., Calis F., Akkaya E., Baran O., Cevik S., Katar S., Gurevin E.G., Hanimoglu H., Hatiboglu M.A., Armutak E.I. (2020). The effect of high mobility group box-1 protein on cerebral edema, blood-brain barrier, oxidative stress and apoptosis in an experimental traumatic brain injury model. Brain Res. Bull..

[61] Aneja R.K., Alcamo A.M., Cummings J., Vagni V., Feldman K., Wang Q., Dixon C.E., Billiar T.R., Kochanek P.M. (2019). Lack of benefit on brain edema, blood–brain barrier permeability, or cognitive outcome in global inducible high mobility group box 1 knockout mice despite tissue sparing after experimental traumatic brain injury. J. Neurotrauma.

[62] Kim J.-B., Choi J.S., Yu Y.-M., Nam K., Piao C.-S., Kim S.-W., Lee M.-H., Han P.-L., Park J.-s., Lee J.-K. (2006). HMGB1, a novel cytokine-like mediator linking acute neuronal death and delayed neuroinflammation in the postischemic brain. J. Neurosci..

[63] Nan K., Han Y., Fang Q., Huang C., Yu L., Ge W., Xiang F., Tao Y.-X., Cao H., Li J. (2019). HMGB1 gene silencing inhibits neuroinflammation via down-regulation of NF-κB signaling in primary hippocampal neurons induced by Aβ25–35. Int. Immunopharmacol..

[64] Xiangjin G., Jin X., Banyou M., Gong C., Peiyuan G., Dong W., Weixing H. (2014). Effect of glycyrrhizin on traumatic brain injury in rats and its mechanism. Chin. J. Traumatol..

[65] Simon D.W., Aneja R.K., Alexander H., Bell M.J., Bayır H., Kochanek P.M., Clark R.S. (2018). Minocycline attenuates high mobility group box 1 translocation, microglial activation, and thalamic neurodegeneration after traumatic brain injury in post-natal day 17 rats. J. Neurotrauma.

[66] Wang K.-Y., Yu G.-F., Zhang Z.-Y., Huang Q., Dong X.-Q. (2012). Plasma high-mobility group box 1 levels and prediction of outcome in patients with traumatic brain injury. Clin. Chim. Acta.

[67] Au A.K., Aneja R.K., Bell M.J., Bayir H., Feldman K., Adelson P.D., Fink E.L., Kochanek P.M., Clark R.S. (2012). Cerebrospinal fluid levels of high-mobility group box 1 and cytochrome C predict outcome after pediatric traumatic brain injury. J. Neurotrauma.

[68] Schuette A.J., Barrow D.L. (2013). Epidemiology and long-term mortality in subarachnoid hemorrhage. World Neurosurg..

[69] Bederson J.B., Connolly E.S., Batjer H.H., Dacey R.G., Dion J.E., Diringer M.N., Duldner J.E., Harbaugh R.E., Patel A.B., Rosenwasser R.H. (2009). Guidelines for the management of aneurysmal subarachnoid hemorrhage: A statement for healthcare professionals from a special writing group of the Stroke Council, American Heart Association. Stroke.

[70] Zhao H., Li Y., Chen L., Shen C., Xiao Z., Xu R., Wang J., Luo Y. (2019). HucMSCs-derived miR-206-knockdown exosomes contribute to neuroprotection in subarachnoid hemorrhage induced early brain injury by targeting BDNF. Neuroscience.

[71] Chen J., Chen G., Li J., Qian C., Mo H., Gu C., Yan F., Yan W., Wang L. (2014). Melatonin attenuates inflammatory response-induced brain edema in early brain injury following a subarachnoid hemorrhage: A possible role for the regulation of pro-inflammatory cytokines. J. Pineal Res..

[72] Lai N., Wu D., Liang T., Pan P., Yuan G., Li X., Li H., Shen H., Wang Z., Chen G. (2020). Systemic exosomal miR-193b-3p delivery attenuates neuroinflammation in early brain injury after subarachnoid hemorrhage in mice. J. Neuroinflamm..

[73] Li Y., Wu P., Dai J., Zhang T., Bihl J., Wang C., Liu Y., Shi H. (2019). Inhibition of mTOR Alleviates Early Brain Injury After Subarachnoid Hemorrhage Via Relieving Excessive Mitochondrial Fission. Cell. Mol. Neurobiol..

[74] Sun Q., Wang F., Li W., Li W., Hu Y.-c., Li S., Zhu J.-h., Zhou M., Hang C.-H. (2013). Glycyrrhizic acid confers neuroprotection after subarachnoid hemorrhage via inhibition of high mobility group box-1 protein: A hypothesis for novel therapy of subarachnoid hemorrhage. Med. Hypotheses.

[75] Murakami K., Koide M., Dumont T.M., Russell S.R., Tranmer B.I., Wellman G.C. (2011). Subarachnoid hemorrhage induces gliosis and increased expression of the pro-inflammatory cytokine high mobility group box 1 protein. Transl. Stroke Res..

[76] An J.Y., Pang H.G., Huang T.Q., Song J.N., Li D.D., Zhao Y.L., Ma X.D. (2018). AG490 ameliorates early brain injury via inhibition of JAK2/STAT3-mediated regulation of HMGB1 in subarachnoid hemorrhage. Exp. Ther. Med..

[77] Sun Q., Wu W., Hu Y.-C., Li H., Zhang D., Li S., Li W., Ma B., Zhu J.-H., Zhou M.-L. (2014). Early release of high-mobility group box 1 (HMGB1) from neurons in experimental subarachnoid hemorrhage in vivo and in vitro. J. Neuroinflamm..

[78] Jing C.-H., Wang L., Liu P.-P., Wu C., Ruan D., Chen G. (2012). Autophagy activation is associated with neuroprotection against apoptosis via a mitochondrial pathway in a rat model of subarachnoid hemorrhage. Neuroscience.

[79] Park J.S., Arcaroli J., Yum H.-K., Yang H., Wang H., Yang K.-Y., Choe K.-H., Strassheim D., Pitts T.M., Tracey K.J. (2003). Activation of gene expression in human neutrophils by high mobility group box 1 protein. Am. J. Physiol. Cell Physiol..

[80] Tian X., Sun L., Feng D., Sun Q., Dou Y., Liu C., Zhou F., Li H., Shen H., Wang Z. (2017). HMGB1 promotes neurovascular remodeling via Rage in the late phase of subarachnoid hemorrhage. Brain Res..

[81] Wang L., Zhang Z., Liang L., Wu Y., Zhong J., Sun X. (2019). Anti-high mobility group box-1 antibody attenuated vascular smooth muscle cell phenotypic switching and vascular remodelling after subarachnoid haemorrhage in rats. Neurosci. Lett..

[82] Richard S.A. (2019). Elucidating the novel biomarker and therapeutic potentials of High-mobility group box 1 in Subarachnoid hemorrhage: A review. AIMS Neurosci..

[83] Sun X., Ji C., Hu T., Wang Z., Chen G. (2013). Tamoxifen as an effective neuroprotectant against early brain injury and learning deficits induced by subarachnoid hemorrhage: Possible involvement of inflammatory signaling. J. Neuroinflamm..

[84] Qiu J., Nishimura M., Wang Y., Sims J.R., Qiu S., Savitz S.I., Salomone S., Moskowitz M.A. (2008). Early release of HMGB-1 from neurons after the onset of brain ischemia. J. Cereb. Blood Flow Metab..

[85] Ieong C., Sun H., Wang Q., Ma J. (2018). Glycyrrhizin suppresses the expressions of HMGB1 and ameliorates inflammative effect after acute subarachnoid hemorrhage in rat model. J. Clin. Neurosci..

[86] Zhang X., Lu Y., Wu Q., Dai H., Li W., Lv S., Zhou X., Zhang X., Hang C., Wang J. (2019). Astaxanthin mitigates subarachnoid hemorrhage injury primarily by increasing sirtuin 1 and inhibiting the Toll-like receptor 4 signaling pathway. FASEB J..

[87] Li Y., Sun F., Jing Z., Wang X., Hua X., Wan L. (2017). Glycyrrhizic acid exerts anti-inflammatory effect to improve cerebral vasospasm secondary to subarachnoid hemorrhage in a rat model. Neurol. Res..

[88] Zhu X.-D., Chen J.-S., Zhou F., Liu Q.-C., Chen G., Zhang J.-M. (2012). Relationship between plasma high mobility group box-1 protein levels and clinical outcomes of aneurysmal subarachnoid hemorrhage. J. Neuroinflamm..

[89] Kiiski H., Långsjö J., Tenhunen J., Ala-Peijari M., Huhtala H., Hämäläinen M., Moilanen E., Öhman J., Peltola J. (2017). Time-courses of plasma IL-6 and HMGB-1 reflect initial severity of clinical presentation but do not predict poor neurologic outcome following subarachnoid hemorrhage. Eneurologicalsci.

[90] Chaudhry S.R., Güresir A., Stoffel-Wagner B., Fimmers R., Kinfe T.M., Dietrich D., Lamprecht A., Vatter H., Güresir E., Muhammad S. (2018). Systemic high-mobility group box-1: A novel predictive biomarker for cerebral vasospasm in aneurysmal subarachnoid hemorrhage. Crit. Care Med..

[91] Wang K.-C., Tang S.-C., Lee J.-E., Li Y.-I., Huang Y.-S., Yang W.-S., Jeng J.-S., Arumugam T.V., Tu Y.-K. (2017). Cerebrospinal fluid high mobility group box 1 is associated with neuronal death in subarachnoid hemorrhage. J. Cereb. Blood Flow Metab..

[92] Hendrix P., Foreman P.M., Harrigan M.R., Fisher W.S., Vyas N.A., Lipsky R.H., Lin M., Walters B.C., Tubbs R.S., Shoja M.M. (2017). Impact of high-mobility group box 1 polymorphism on delayed cerebral ischemia after aneurysmal subarachnoid hemorrhage. World Neurosurg..

[93] Sokół B., Woźniak A., Jankowski R., Jurga S., Wąsik N., Shahid H., Grześkowiak B. (2015). HMGB1 level in cerebrospinal fluid as a marker of treatment outcome in patients with acute hydrocephalus following aneurysmal subarachnoid hemorrhage. J. Stroke Cerebrovasc. Dis..

[94] King M.D., Laird M.D., Ramesh S.S., Youssef P., Shakir B., Vender J.R., Alleyne C.H., Dhandapani K.M. (2010). Elucidating novel mechanisms of brain injury following subarachnoid hemorrhage: An emerging role for neuroproteomics. Neurosurg. Focus.

[95] Nakahara T., Tsuruta R., Kaneko T., Yamashita S., Fujita M., Kasaoka S., Hashiguchi T., Suzuki M., Maruyama I., Maekawa T. (2009). High-mobility group box 1 protein in CSF of patients with subarachnoid hemorrhage. Neurocrit. Care.

[96] Uzawa A., Mori M., Taniguchi J., Masuda S., Muto M., Kuwabara S. (2013). Anti-high mobility group box 1 monoclonal antibody ameliorates experimental autoimmune encephalomyelitis. Clin. Exp. Immunol..

[97] Zhao J., Wang Y., Xu C., Liu K., Wang Y., Chen L., Wu X., Gao F., Guo Y., Zhu J. (2017). Therapeutic potential of an anti-high mobility group box-1 monoclonal antibody in epilepsy. Brain. Behav. Immun..

[98] Mollica L., De Marchis F., Spitaleri A., Dallacosta C., Pennacchini D., Zamai M., Agresti A., Trisciuoglio L., Musco G., Bianchi M.E. (2007). Glycyrrhizin binds to high-mobility group box 1 protein and inhibits its cytokine activities. Chem. Biol..

[99] Davé S.H., Tilstra J.S., Matsuoka K., Li F., DeMarco R.A., Beer-Stolz D., Sepulveda A.R., Fink M.P., Lotze M.T., Plevy S.E. (2009). Ethyl pyruvate decreases HMGB1 release and ameliorates murine colitis. J. Leukoc. Biol..

[100] Nishibori M., Mori S., Takahashi H.K. (2019). Anti-HMGB1 monoclonal antibody therapy for a wide range of CNS and PNS diseases. J. Pharmacol. Sci..

[101] Paudel Y.N., Angelopoulou E., Semple B., Piperi C., Othman I., Shaikh M.F. (2020). Potential neuroprotective effect of the HMGB1 inhibitor Glycyrrhizin in neurological disorders. ACS Chem. Neurosci..

[102] Okuma Y., Wake H., Teshigawara K., Takahashi Y., Hishikawa T., Yasuhara T., Mori S., Takahashi H.K., Date I., Nishibori M. (2019). Anti–High Mobility Group Box 1 Antibody Therapy May Prevent Cognitive Dysfunction After Traumatic Brain Injury. World Neurosurg..

[103] Wang D., Liu K., Wake H., Teshigawara K., Mori S., Nishibori M. (2017). Anti-high mobility group box-1 (HMGB1) antibody inhibits hemorrhage-induced brain injury and improved neurological deficits in rats. Sci. Rep..

[104] Qiu X., Cheng X., Zhang J., Yuan C., Zhao M., Yang X. (2020). Ethyl pyruvate confers protection against endotoxemia and sepsis by inhibiting caspase-11-dependent cell pyroptosis. Int. Immunopharmacol..

[105] Zhang T., Guan X.-W., Gribben J.G., Liu F.-T., Jia L. (2019). Blockade of HMGB1 signaling pathway by ethyl pyruvate inhibits tumor growth in diffuse large B-cell lymphoma. Cell Death Dis..

[106] Bhat S.M., Massey N., Karriker L.A., Singh B., Charavaryamath C. (2019). Ethyl pyruvate reduces organic dust-induced airway inflammation by targeting HMGB1-RAGE signaling. Respir. Res..

[107] Ji J., Fu T., Dong C., Zhu W., Yang J., Kong X., Zhang Z., Bao Y., Zhao R., Ge X. (2019). Targeting HMGB1 by ethyl pyruvate ameliorates systemic lupus erythematosus and reverses the senescent phenotype of bone marrow-mesenchymal stem cells. Aging.

[108] Su X., Wang H., Zhao J., Pan H., Mao L. (2011). Beneficial effects of ethyl pyruvate through inhibiting high-mobility group box 1 expression and TLR4/NF-B pathway after traumatic brain injury in the rat. Mediat. Inflamm..

[109] Calder P.C. (2017). Omega-3 fatty acids and inflammatory processes: From molecules to man. Biochem. Soc. Trans..

[110] Bailes J.E., Abusuwwa R., Arshad M., Chowdhry S.A., Schleicher D., Hempeck N., Gandhi Y.N., Jaffa Z., Bokhari F., Karahalios D. (2020). Omega-3 fatty acid supplementation in severe brain trauma: Case for a large multicenter trial. J. Neurosurg..

[111] Chen X., Wu S., Chen C., Xie B., Fang Z., Hu W., Chen J., Fu H., He H. (2017). Omega-3 polyunsaturated fatty acid supplementation attenuates microglial-induced inflammation by inhibiting the HMGB1/TLR4/NF-κB pathway following experimental traumatic brain injury. J. Neuroinflamm..

[112] Descotes J. (2009). Immunotoxicity of monoclonal antibodies. MAbs.

[113] Ohnishi M., Katsuki H., Fukutomi C., Takahashi M., Motomura M., Fukunaga M., Matsuoka Y., Isohama Y., Izumi Y., Kume T. (2011). HMGB1 inhibitor glycyrrhizin attenuates intracerebral hemorrhage-induced injury in rats. Neuropharmacology.

[114] Pang H., Huang T., Song J., Li D., Zhao Y., Ma X. (2016). Inhibiting HMGB1 with glycyrrhizic acid protects brain injury after DAI via its anti-inflammatory effect. Mediat. Inflamm..

[115] Sehba F.A., Hou J., Pluta R.M., Zhang J.H. (2012). The importance of early brain injury after subarachnoid hemorrhage. Prog. Neurobiol..

[116] Zhang X.-S., Li W., Wu Q., Wu L.-Y., Ye Z.-N., Liu J.-P., Zhuang Z., Zhou M.-L., Zhang X., Hang C.-H. (2016). Resveratrol attenuates acute inflammatory injury in experimental subarachnoid hemorrhage in rats via inhibition of TLR4 pathway. Int. J. Mol. Sci..

[117] Chang C.-Z., Lin C.-L., Wu S.-C., Kwan A.-L. (2014). Purpurogallin, a natural phenol, attenuates high-mobility group box 1 in subarachnoid hemorrhage induced vasospasm in a rat model. Int. J. Vasc. Med..

[118] Chang C.-Z., Wu S.-C., Kwan A.-L., Lin C.-L. (2016). Rhinacanthin-C, a fat-soluble extract from Rhinacanthus nasutus, modulates high-mobility group box 1-related neuro-inflammation and subarachnoid hemorrhage-induced brain apoptosis in a rat model. World Neurosurg..

[119] Wang Z., Wu L., You W., Ji C., Chen G. (2013). Melatonin alleviates secondary brain damage and neurobehavioral dysfunction after experimental subarachnoid hemorrhage: Possible involvement of TLR 4-mediated inflammatory pathway. J. Pineal Res..

[120] Chang C.-Z., Wu S.-C., Kwan A.-L. (2015). Glycyrrhizin attenuates proinflammatory cytokines through a peroxisome proliferator-activated receptor-γ-dependent mechanism and experimental vasospasm in a rat model. J. Vasc. Res..

[121] Chang C.-Z., Wu S.-C., Kwan A.-L., Lin C.-L. (2015). 4′-O-β-d-glucosyl-5-O-methylvisamminol, an active ingredient of Saposhnikovia divaricata, attenuates high-mobility group box 1 and subarachnoid hemorrhage-induced vasospasm in a rat model. Behav. Brain Funct..

[122] Yang H., Tracey K.J. (2010). Targeting HMGB1 in inflammation. Biochim. Biophys. Acta (BBA) Gene Regul. Mech..

